# Comparative Benchmarking of Glass and Silicon Nitride Nanopores for Single-Molecule Detection

**DOI:** 10.1021/acsnano.6c03089

**Published:** 2026-05-05

**Authors:** Fei Zheng, Zhan Wang, An Bai, Rui Hu, Xianhu Sun, Jingjie Sha, Qing Zhao, Kaikai Chen, Ulrich F. Keyser

**Affiliations:** † School of Nanoscience and Nanotechnology, 74519 University of Chinese Academy of Sciences, Beijing 101408, China; ‡ Cavendish Laboratory, University of Cambridge, Cambridge CB3 0US, United Kingdom; § State Key Lab for Mesoscopic Physics and Frontiers Science Center for Nano-Optoelectronics, Electron Microscopy Laboratory, School of Physics, 12465 Peking University, Beijing 100871, China; ∥ School of Chemical Sciences, 74519 University of Chinese Academy of Sciences, Beijing 101408, China; ⊥ Jiangsu Key Laboratory for Design and Manufacture of Precision Medicine Equipment, School of Mechanical Engineering, 12579 Southeast University, Nanjing 211189, China

**Keywords:** glass nanopore, SiN_*x*_ nanopore, RMS noise, signal-to-noise ratio, translocation dynamics

## Abstract

In the rapidly evolving field of single-molecule sensing, solid-state nanopores have emerged as transformative tools for the label-free detection of biomolecules, ranging from DNA polymers to proteins. Yet, with two dominant platforms—glass nanopores and silicon nitride (SiN_*x*_) nanopores—researchers face a pivotal choice: which architecture best unlocks superior performance? Here, we deliver a head-to-head experimental comparison under comparable experimental conditions, benchmarking noise characteristics, signal-to-noise ratios (SNRs), and translocation dynamics for DNA and protein analytes across matched nanopore sizes. Our findings reveal compelling trade-offs: glass nanopores excel in DNA sensing, achieving record SNR values of >80 in 5 nm nanopores (4 M LiCl, 50 kHz filter cutoff) due to their conical geometry that focuses electric fields. In contrast, SiN_*x*_ nanopores dominate protein detection with SNR values of >120, leveraging thin membranes for enhanced current blockade from volume exclusion. Comprehensive performance metrics—including unfolded DNA fraction, backward-to-forward translocation time ratio, translocation frequency, and perturbed events—also show distinct translocation behaviors of biomolecules in the two nanopore platforms. These insights, supported by finite-element simulations, establish a mechanistic framework for nanopore selection, favoring conical glass nanopores for polymeric analytes and SiN_*x*_ membrane nanopores for compact biomolecules. This work not only sets benchmarks for nanopore sensitivity but also enables the development of tailored sensors in diagnostics, sequencing, and beyond, advancing nanotechnology for high-resolution biomolecular analyses.

## Graphical abstract

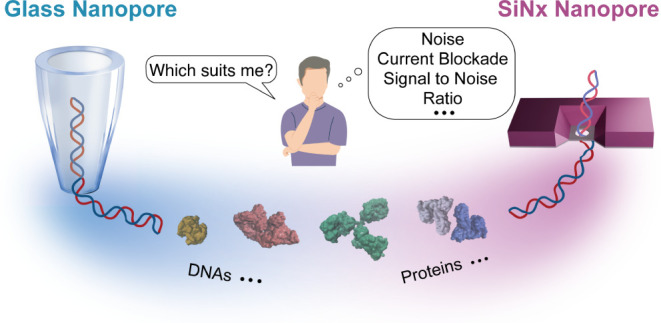

## Introduction

Nanopore sensing has progressed from a promising idea with applications in single-molecule biophysics to a mainstream analytical platform in just over two decades. While biological nanopores were the first to demonstrate single-molecule detection,^1^ solid-state nanopores have also rapidly emerged as powerful tools. Solid-state nanopores offer distinct advantages, including enhanced robustness, tunability of pore dimensions, and compatibility with microfabrication techniques, which expand both their operating conditions and analytical applications.^2–6^ Among the myriad solid-state implementations explored since, two families, “glass nanopores” ^2,7,8^ and “silicon-nitride (SiN_*x*_) nanopores”,^2,9–11^ have matured into reliable sensors, outperforming their counterparts. Each embodies a distinct fabrication philosophy: glass nanopores exploit rapid, direct fabrication by pulling quartz capillaries, while SiN_*x*_ nanopores harness lithographic patterning to create ultrathin membranes with precisely located apertures. Both nanopore platforms enable label-free detection of single DNA/RNA polymers,^12–16^ proteins,^8,17–21^ nanoparticles,^22–25^ or even entire viruses^26,27^ by monitoring ionic current blockades as analytes translocate through a nanoscale orifice. Yet, despite dozens of studies reporting ever-increasing bandwidths,^28–31^ signal-to-noise ratios (SNR),^14,32–35^ and application niches,^17,18,36–40^ a systematic comparison of nanopore performance depending on the nanopore platform is still lacking. The charge and shape of the analyte molecule may influence when a researcher or an instrument designer should favor one nanopore architecture over the other. The absence of such a comparative map is increasingly conspicuous as nanopore technology migrates from proof-of-concept demonstrations in academic laboratories to deployed diagnostic and sequencing devices that must meet stringent performance, cost, and manufacturability requirements.

The popularity of glass nanopores stems from their straightforward production: a laser puller elongates a quartz capillary until its tip necks down to a few nanometers, creating an aperture that serves directly as the sensing pore. Laser pulling enables more rapid, lower-cost fabrication of nanopores with low electric noise compared to SiN_*x*_ nanopores,^2,6,7^ making glass nanopores very attractive for sensing experiments. Beyond fabrication simplicity, the conical geometry inherently funnels ionic current, enabling asymmetric transport of ions and molecules^41^ within the nanopore tip, which results in ion current rectification (ICR)^42–44^ that can be exploited for selective sensing,^45,46^ analyte discrimination,^47–49^ and ionic gating.^50,51^ Moreover, the conical geometry and mechanical rigidity of glass nanopores make them ideal probes for scanning probe microscopy applications, enabling high-resolution control of the nanopore displacement.^33,52^ Nevertheless, glass nanopores have trade-offs. A key challenge is the reproducible fabrication and subsequent filling of pores smaller than 5 nm.^2,53^ For applications requiring multiple pores, multibarrel capillaries allow fabrication of double or quad nanopore arrays,^54,55^ but scaling beyond this remains challenging. The elongated geometry of glass nanopores should also result in more extended electric fields and, hence, a larger sensing volume compared with SiN_*x*_ nanopores. As a result, the current blockades generated by analyte passage, as well as SNR, may be lower depending on the shape of the molecule and, hence, limit detection sensitivity.

SiN_*x*_ nanopores, in contrast, leverage the precision of semiconductor micro- and nanofabrication: silicon nitride films are deposited on silicon wafers, patterned lithographically, and back-etched to create freestanding membranes merely 10–30 nm thick, with individual pores subsequently drilled at predetermined locations using beam-based technologies^2,9,56^ or formed by controlled dielectric breakdown.^3,57^ Both techniques provide fine-tuned control over nanopore diameter and length, where the ultrathin membrane enhances sensing resolution by confining the detection volume. Equally important, the compatibility of SiN_*x*_ nanopores with semiconductor fabrication workflows opens pathways for sophisticated device integration,^58–61^ from on-chip electronics to microfluidic systems. Furthermore, SiN_*x*_ nanopores are particularly well-suited for high-throughput applications requiring parallel sensing channels,^62–64^ such as DNA sequencing, protein analysis, and viral particle characterization. Yet, this technological sophistication comes with barriers: the fabrication process requires expensive instrumentation and specialized expertise,^2,65,66^ while production yields for sensing-grade nanopores remain suboptimal.^57^ Additionally, the thin SiN_*x*_ membranes are prone to damage during chemical cleaning or handling, and nanopore sizes may change when moving from vacuum to aqueous conditions.^2^

Existing views of these two nanopore platforms emphasize fabrication workflow and cost. Yet, more fundamentally, their contrasting characteristics leave researchers with a critical question: which nanopore best serves their particular sensing application (Figure 1A)? Here, we address this decision-making challenge through direct experimental comparison under equivalent conditions.

**Figure 1:**
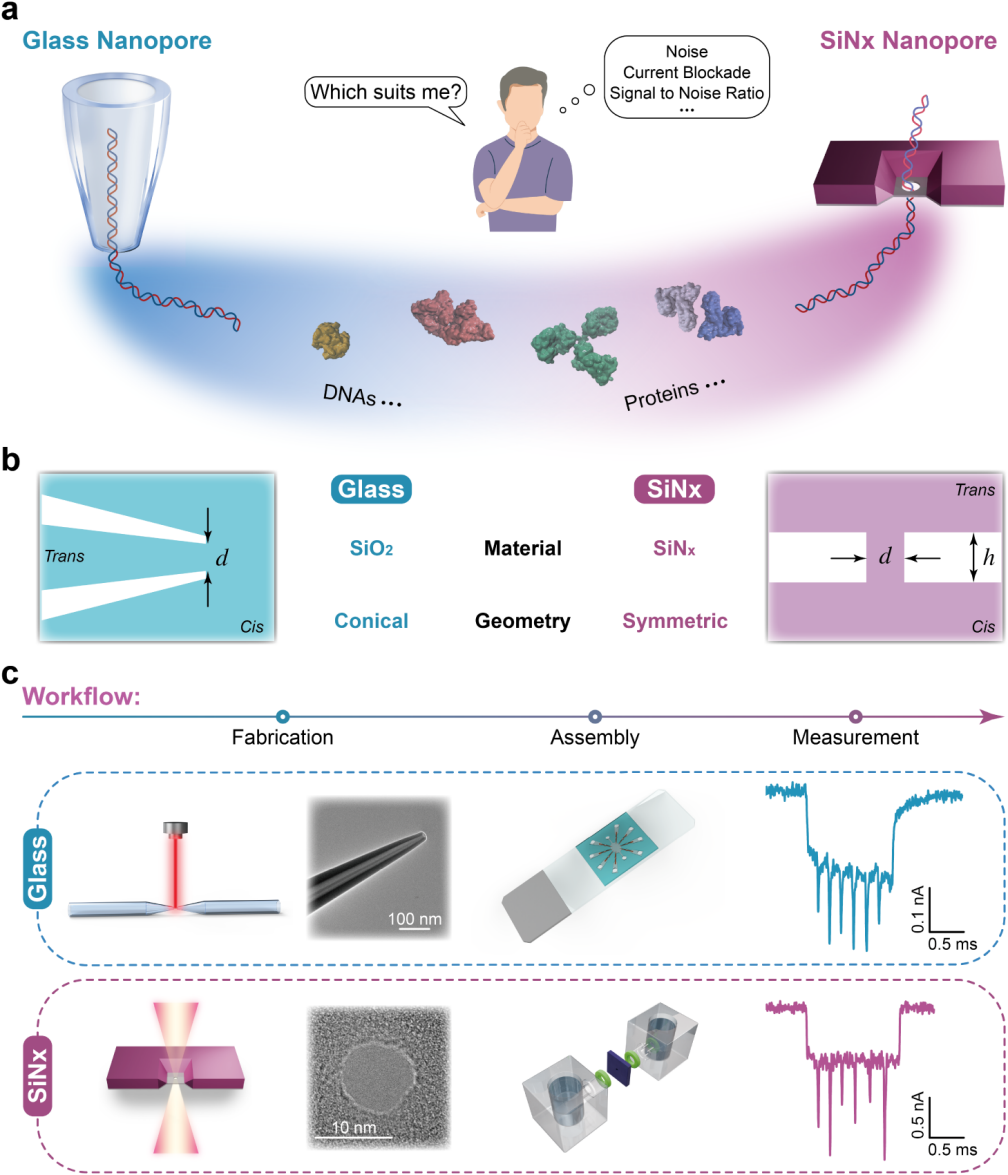
Comparison of glass and SiN*_x_* nanopore platforms for biomolecular detection. (a) Conceptual illustration showing the two nanopore types: glass nanopores (left) and SiN*_x_* nanopores (right), used for DNA and protein sensing. (b) Schematic illustrating the key differences between the two nanopore platforms. The nanopore size parameters *d* and *h,* are indicated. The *cis* side of the SiN*_x_* nanopore corresponds to the locally thinned region, whereas the *trans* side corresponds to the trapezoidal structure (see Supplementary Figure S2). (c) Experimental workflow for nanopore fabrication, assembly, and measurement. Top panel: Glass nanopores were fabricated by laser-assisted pulling of quartz capillaries. Bottom panel: SiN*_x_* nanopores were created by focused electron-beam drilling in a TEM on free-standing SiN*_x_* membranes. Representative TEM images show nanopores with matched diameters (∼10 nm). Each nanopore was integrated into a custom microfluidic assembly for ionic current recording. Example ionic current traces of DNA translocation events are shown on the right.

## Results/Discussion

### Workflow of the Two Nanopore Platforms

Figure 1b summarizes the key differences between the two nanopore platforms in terms of their material and geometry. The glass nanopore is composed of silicon oxide (quartz), whereas the SiN_*x*_ nanopore is made of silicon nitride. The glass nanopore exhibits an asymmetrically conical shape from the *cis* to *trans* side, while the SiN_*x*_ nanopore is nearly symmetric, typically displaying a cylindrical or hourglass-like profile depending on the fabrication beam parameters.^67^

We fabricated the nanopores of two types using the following materials and workflows. For the glass platform (Figure 1c, top), quartz capillaries (SiO_2_; outer diameter 0.5 mm, inner diameter 0.2 mm) were processed by laser-assisted pulling to form nanopores with a diameter *d* (Figure 1b). Pulling parameters and the corresponding pore diameters are summarized in Supplementary Table S1; note that optimal parameters may vary slightly with laboratory conditions (temperature and humidity). For the SiN_*x*_ platform (Figure 1c, bottom), nanopores were created in free-standing SiN_*x*_ membranes by electron-beam drilling in a transmission electron microscope (TEM). The MEMS process flow used to fabricate the SiN_*x*_ membrane chips is provided in Supplementary Note 1 and Figure S1. Each chip contains a 20 × 20 μm^2^ suspended SiN_*x*_ window with a locally thinned region (diameter ϕ = 2.5 μm; thickness *h* ∼ 15 nm) where the nanopore is positioned. A 1-μm SiO_2_ layer between the SiN_*x*_ membrane and the Si substrate serves as an additional dielectric, reducing chip capacitance^34,68^ (Supplementary Figure S2). The fabricated SiN_*x*_ nanopores have a diameter *d* and a length *h* (Figure 1b).

Nanopore diameters were matched across platforms (same diameter, *d*; Figure 1b). For SiN_*x*_ nanopores, this is the diameter of the constriction drilled into the membrane. For glass nanopores, this is the inner diameter at the tip of the quartz capillary. Representative TEM images of two 10 nm nanopores are shown in Figure 1c. Nanopores were then integrated into custom microfluidic assemblies (Figure 1c). A PDMS layer at the *cis*–*trans* interface in the glass nanopore device and two PDMS gaskets clamping the SiN_*x*_ chip between the *cis* and *trans* fluidic cells were employed to reduce the effective chip area exposed to the electrolyte and thereby decrease electrical noise in the recordings.^34,69^

To benchmark performance, we measured DNA and protein analytes (Figure 1a). Example traces for a DNA construct carrying six barcoded markers are shown in Figure 1c. All measurements were performed in 4 M LiCl, 10 mM Tris-EDTA (pH 9, adjusted with LiOH). Data were sampled at 1 MHz and low-pass filtered at 50 kHz for the ionic current and 5 kHz for the voltage. Additional experimental details (fabrication of glass and SiN_*x*_ nanopores and nanopore measurements) are provided in Supplementary Notes 2–4.

### Nanopore Noise and Electric Field Comparison

We first compared the noise performance of the two nanopore platforms by quantifying the root-mean-square (RMS) current noise. Figure 2a presents representative open-pore current traces at 0 mV for a glass and a SiN_*x*_ nanopore with matched diameters, as confirmed by TEM imaging (Figure 1b) and current–voltage (I–V) characterization (Figure 2b and c). The SiN_*x*_ nanopore exhibits a noise amplitude approximately four times higher than that of the glass nanopore (Figure 2a). We then evaluated RMS noise across batches of glass and SiN_*x*_ nanopores (N = 10), with each value calculated from a 3-s current trace under identical filtering conditions (Figure 2d). Because the low-pass filter cutoff strongly influences RMS estimates, we used a cutoff frequency of 50 kHz, higher than the commonly used 10 kHz, to enable a broader recording bandwidth. Across devices, the average RMS noise, < RMS_*n*_>, was 5.8 pA for glass and 29.7 pA for SiN_*x*_. The power spectral densities (PSDs) of the two platforms are shown in Figure 2i and j. In the frequency range of 10^3^–10^5^ Hz, the PSD of the SiN_*x*_ nanopore is ∼10^–1^ pA^2^/Hz, compared with ∼10^–3^ pA^2^/Hz for the glass nanopore. This frequency range is dominated by dielectric and capacitive noise, which scale with chip capacitance;^32^ the lower permittivity and thicker insulating layer of the glass nanopores reduce capacitance and thereby suppress high-frequency noise. We also examined the dependence of RMS noise on nanopore diameter (Figure 2k and l). For both platforms, RMS noise increases approximately linearly over the range up to 20 nm, consistent with thermal noise, which is inversely proportional to pore resistance. In addition, RMS noise exhibits a quadratic dependence on applied voltage (Supplementary Figure S3): for a 10 nm glass nanopore, it increases from 5.8 pA at 0 mV to 6.1 pA at ±600 mV, and for a SiN_*x*_ nanopore from 26.1 pA at 0 mV to 31–32 pA at ±130 mV, all for a cutoff frequency of 50 kHz (the voltage ranges for both nanopores are selected based on the “comparable experimental conditions” discussed later).

**Figure 2:**
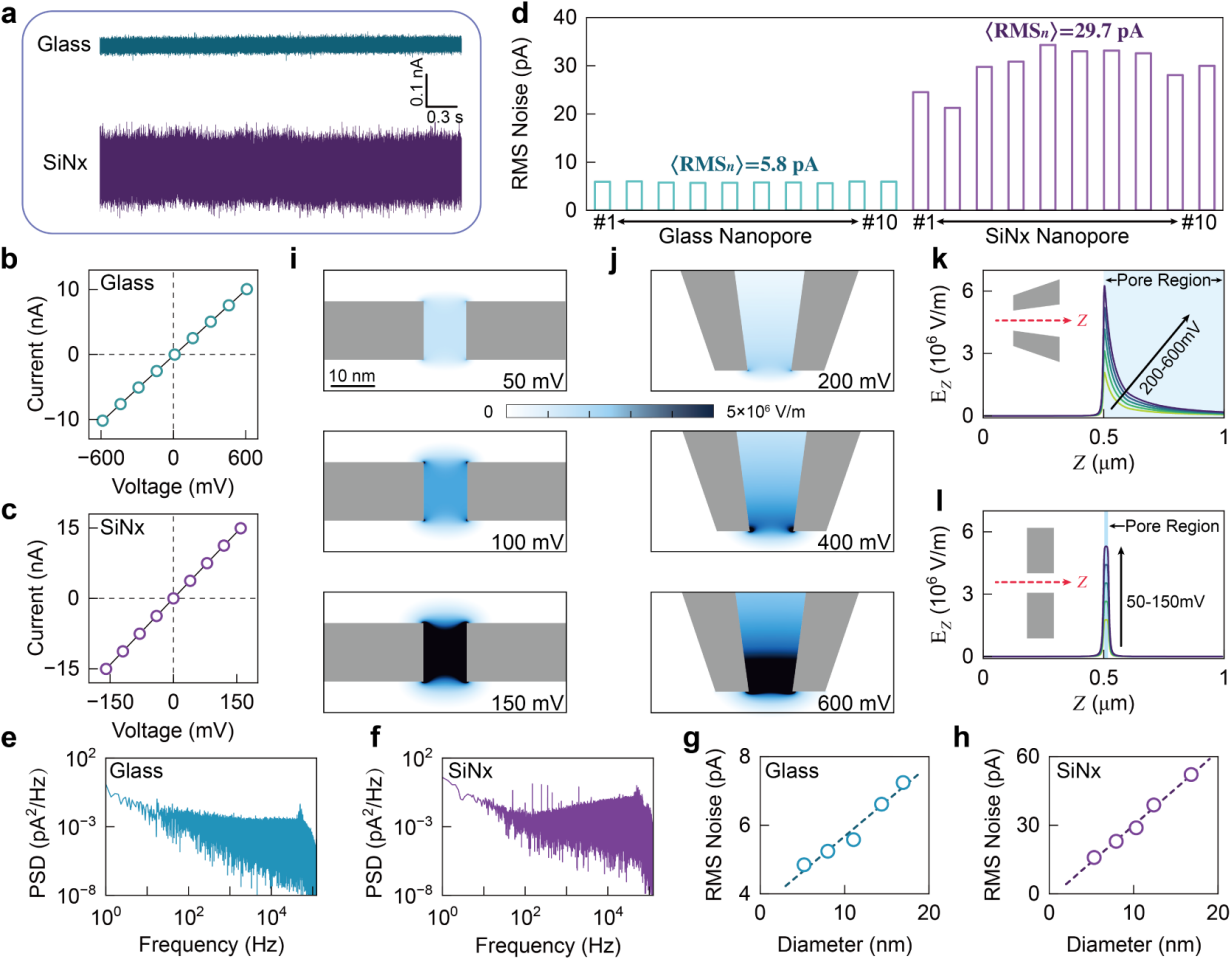
Noise characterization and electric field distribution of glass and SiN*_x_* nanopores. (a) Representative open-pore current traces recorded at 0 mV for glass and SiN*_x_* nanopores of identical diameter, with a sampling rate of 1 MHz and a cutoff frequency of 50 kHz. (b,c) I–V characteristics of glass and SiN*_x_* nanopores, respectively. (d) RMS current noise values of glass and SiN*_x_* nanopores (N = 10 for each), calculated from 3-s open-pore traces filtered at 50 kHz, yielding average RMS noise values of ⟨RMS_n_⟩ = 5.8 pA (glass) and 29.7 pA (SiN*_x_*). (e,f) PSD plots of the open-pore current noise for glass and SiN*_x_* nanopores, respectively, measured at 0 mV with a 50 kHz cutoff frequency. (g,h) Dependence of RMS noise on nanopore diameter (0–20 nm) for glass and SiN*_x_* nanopores. Each point is the average value measured from three independent nanopores. The buffer is 4 M LiCl, 10 mM Tris-EDTA (pH 9). (i,j) FEM simulations showing electric field distributions in the glass and SiN*_x_* nanopores at the indicated applied voltages, with both devices modeled at a 10 nm diameter. (k,l) Axial electric field profiles along the nanopore axis for glass (200–600 mV) and SiN*_x_* (50–150 mV) nanopores, respectively.

To ensure a meaningful comparison between the two nanopore platforms, we evaluated not only their pore diameters but also the electric field distributions near the pore entrance. Finite element method (FEM) simulations were performed to characterize the electric field distribution in glass and SiN_*x*_ nanopores under different applied voltages. The corresponding simulation models are shown in Supplementary Figure S4 and Note 7. Both nanopores were modeled with a diameter of 10 nm, while the geometric parameters of the glass nanopore, including the cone angle and axial length, were derived from previous experimental characterizations.^13,70^ Figure 2e and f present the simulated electric field maps for three representative voltages in the glass and SiN_*x*_ nanopores, respectively. The results indicate that a substantially higher voltage is required for the glass nanopore to achieve the same peak electric field strength as that in the SiN_*x*_ nanopore of identical diameter. Specifically, applied potentials of 50, 100, and 150 mV in the SiN_*x*_ nanopore correspond approximately to 200, 400, and 600 mV in the glass nanopore, respectively. This relationship is further evidenced by the axial electric field profiles along the nanopore axis (Figures 2g and h). For instance, at 400 mV, a glass nanopore exhibits a peak field strength of 4.1 × 10^6^ V·m^–1^, which is comparable to 3.5 × 10^6^ V·m^–1^ observed at 100 mV in the SiN_*x*_ nanopore. While the SiN_*x*_ nanopore displays a relatively uniform electric field distribution, the glass nanopore features a gradually decaying field along its conical section, forming a long tail extending roughly 500 nm into the pore interior (enlarged views of the electric field strength profiles are provided in Supplementary Figure S5).

Interestingly, despite exhibiting similar peak field strengths, the two nanopore platforms yielded distinct biomolecular translocation behaviors. As shown in Supplementary Figure S6, double-stranded DNA molecules (dsDNA, 7.2 kbp) displayed a median translocation time of 2.16 ms at 400 mV in the glass nanopore, nearly twice that measured at 100 mV in the SiN_*x*_ nanopore (1.1 ms). This difference may stem from the gradually decreasing electric field along the conical geometry of the glass nanopores. Instead, the translocation speed at 500 mV in glass nanopores closely matched that at 100 mV in SiN_*x*_ nanopores, yielding a median dwell time of 1.06 ms (Supplementary Figure S6). Consequently, we define “comparable experimental conditions” in this study based on matched median dwell times as our driving-force proxy, utilizing applied voltages of 500 mV for glass nanopores and 100 mV for SiN_*x*_ nanopores as a consistent basis for all subsequent benchmarking. By standardizing the temporal scale of analyte transport, this approach allows us to evaluate the spatial detection capabilities of each platform, specifically current blockade and SNR.

### Comparison of Sensing DNA

Next, we compared DNA sensing performance between glass and SiN_*x*_ nanopore platforms by measuring three DNA species: double-stranded M13mp18 DNA (7.2 kbp), topological variants of λ-DNA (knots and plectonemes), and a designed DNA construct. The construct comprises six evenly spaced groups of dumbbell motifs attached to the scaffold, with eight dumbbells per site (Figure 3c), and was assembled by hybridizing complementary oligonucleotides to a single-stranded M13mp18 scaffold (see Supplementary Note 5 for details). Representative translocation signals for all three species in glass and SiN_*x*_ nanopores are shown in Figure 3a–c; both platforms resolve fine DNA nanostructures and topological features (see Supplementary Figures S7–S10 for additional examples). Unless noted otherwise, these measurements were performed at 500 mV (glass) and 100 mV (SiN_*x*_). Compared with glass pores, SiN_*x*_ pores produce larger current blockades across these cases. All-points current histograms for linear dsDNA translocations (excluding folded events) in 10 nm pores (Figure 3d) yield mean blockade currents of 0.50 nA for SiN_*x*_ and 0.12 nA for glass (∼4× difference). The full width at half-maximum (fwhm) of the open-pore baseline is greater for SiN_*x*_ than for glass (Figure 3d), again indicating higher noise in SiN_*x*_ nanopores. Moreover, in glass nanopores, the blockade fwhm is comparable to the baseline fwhm, whereas in SiN_*x*_ nanopores, the blockade distribution is substantially broader than the baseline. Figure 3e summarizes the mean current blockade, Δ̅I, for a set of pores (means from the histogram fits), again showing larger Δ̅IΔ̅I for the three SiN_*x*_ nanopores than for glass. Using SNR = Δ̅I/RMS noise, the resulting SNRs for the 10 nm pores (Figure 3f) are similar for both platforms (∼20), indicating comparable effective sensitivity for DNA detection at this pore size.

**Figure 3:**
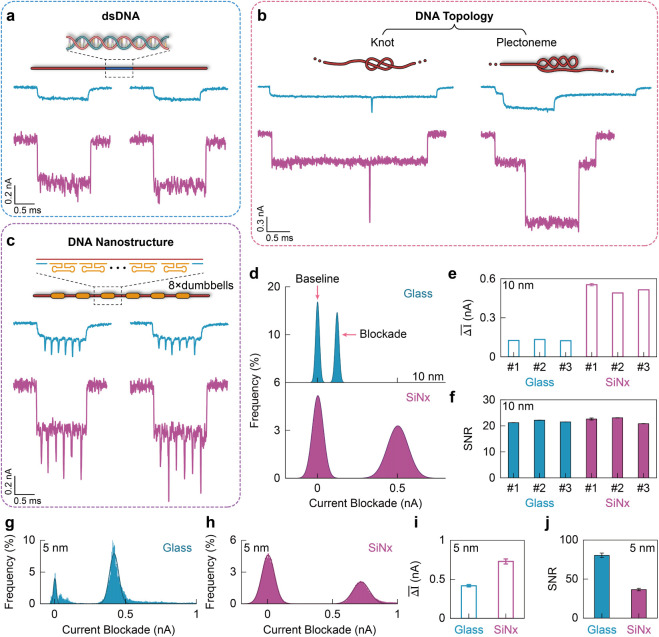
Comparison of DNA signal-to-noise performance in glass and SiN*_x_* nanopores. (a–c) Representative translocation event signals of three DNA species: (a) double-stranded M13mp18 DNA (7.2 kbp), (b) topological λ-DNA variants (knot and plectoneme), and (c) a designed DNA construct containing six evenly spaced groups of eight dumbbell motifs. (d) All-points current histograms of linear dsDNA translocations in 10 nm nanopores, showing baseline and blockade current levels for glass (top) and SiN*_x_* (bottom). See Supplementary Figure S22 for histograms of other DNA types. (e) Summary of mean current blockade values (Δ̅I) for three independent nanopores of each platform at 10 nm diameter. (f) Corresponding SNRs showing comparable sensing performance between platforms at 10 nm. Error bars represent the standard deviation (SD) obtained from the Gaussian fitting of the current blockade distribution. (g, h) All-points current histograms for DNA translocations through 5 nm glass (g) and SiN*_x_* (h) nanopores. (i) Comparison of mean current blockades for both platforms at 5 nm, derived from the histograms. (j) Corresponding SNRs for 5 nm nanopores, highlighting that glass nanopores exhibit a larger increase in DNA sensitivity upon pore size reduction. Error bars represent SDs from measurements on three independent nanopores. Note that in some cases, the error bars are too small to be visible. Measurements were performed at 500 mV (glass) and 100 mV (SiN*_x_*). The sampling rate is 1 MHz, and the cutoff frequency is 50 kHz.

Given the dsDNA double-helix diameter of ∼2.2 nm, we evaluated both platforms using smaller pores (5 nm, characterized by TEM scanning; Supplementary Figure S11). Figure 3g and h show all-points current histograms for linear DNA translocations in a glass and a SiN_*x*_ nanopore, respectively, from which we extracted the mean current blockade Δ̅I (summarized in Figure 3i). In the glass nanopore, Δ̅I increases from 0.12 nA (10 nm) to 0.42 nA (5 nm; 3.5-fold). In SiN_*x*_, Δ̅I increases from 0.50 nA to 0.72 nA (1.44-fold). Notably, the 1.44-fold increase in Δ̅I for SiN_*x*_ nanopores is much smaller than that for glass nanopores (3.5×). By contrast, when reducing the nanopore diameter from 10 to 5 nm, the SNR of SiN_*x*_ increases from ∼20 to ∼36, whereas that of glass rises from ∼20 to ∼81 (Figure 3j). In addition, as the diameter shrinks from 10 to 5 nm, the electric field strength in glass nanopores increases markedly (from ∼4.1 × 10^6^ to ∼8.8 × 10^6^ V·m^–1^ at 400 mV), whereas the change is modest in SiN_*x*_ nanopores (from ∼3.5 × 10^6^ to ∼4.1× 10^6^ V·m^–1^ at 100 mV; Supplementary Figure S14), due to the high aspect ratio of glass nanopores. See Supplementary Figure S23 for the SNR of a medium nanopore size (∼7 nm) for both platforms.

It is important to mention that if the glass nanopore were biased to match the SiN_*x*_ translocation speed (i.e., at higher voltage), the SNR would be even greater because the RMS noise increases only weakly with voltage (Supplementary Figure S3). We also characterized the glass nanopore at 300 and 400 mV (Supplementary Figures S12–S13), yielding mean blockades of 0.27 and 0.35 nA and SNRs of ∼54 and ∼70, respectively, again exceeding that for the SiN_*x*_ nanopore at 100 mV.

Beyond SNR, we evaluated additional factors that determine sensing performance. As summarized in Figure 4a, two primary attributes define a nanopore: geometry and material. These, in turn, set secondary properties such as surface charge density, hydrophilicity, roughness, and mechanical stability, all of which strongly impact device usability and performance. Having assessed the main performance metrics, including baseline RMS noise, current blockade, dwell time, and SNR, we further quantified four additional criteria: folded-event fraction, directional dwell-time asymmetry (τ_backward_/τ_forward_), translocation frequency, and the fraction of “perturbed” events. The durability of glass^71^ and SiN_*x*_^56^ nanopores has been reported previously and is not discussed in this work.

**Figure 4:**
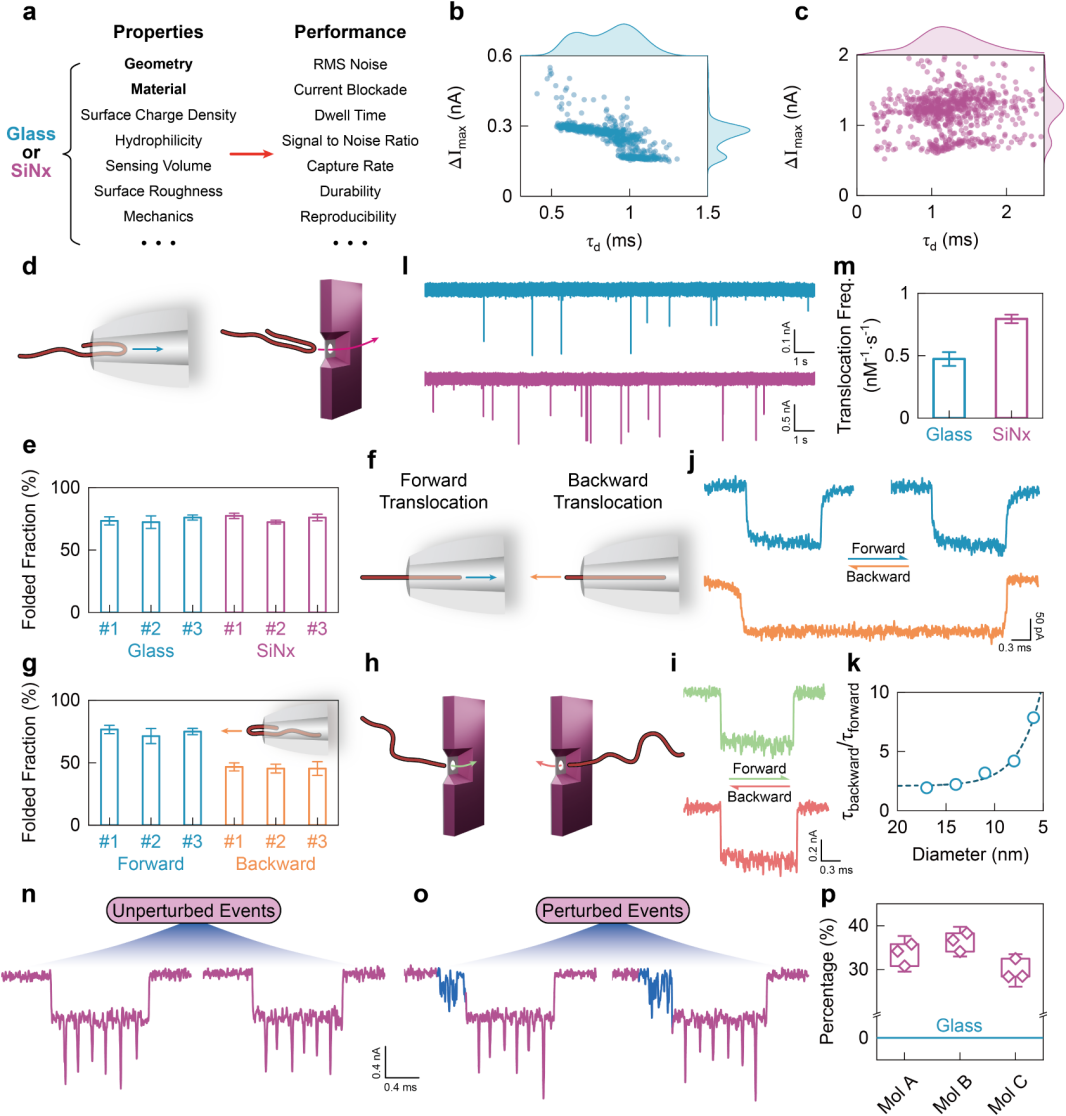
Comparison of DNA translocation dynamics in glass and SiN*_x_* nanopores. (a) Summary of key structural and material properties that define a nanopore and the metrics used to evaluate its sensing performance. (b,c) Representative scatter plots of maximum current blockade (ΔI_max_) versus dwell time (τ_d_) for dsDNA translocation events in glass and SiN*_x_* nanopores, respectively. (d) Schematic illustrating folded translocation through glass and SiN*_x_* nanopores in the forward direction. (e) Folded-event fractions for three independent glass and SiN*_x_* nanopores measured in 4 M LiCl. Error bars represent the SDs from three measurements per nanopore. (f) Schematic illustrating forward and backward translocation directions in a glass nanopore. (g) Comparison of folded-event fractions in conical glass nanopores under forward (tip-to-interior) and backward (interior-to-tip) translocation. Error bars represent the SDs from three measurements per nanopore. (h,i) Schematic illustrating forward and backward translocation directions in a SiN*_x_* nanopore. Example forward and backward translocation events in SiN*_x_* nanopores. (j) Example translocation events showing slower backward translocation (bottom) compared with forward translocation (top) in glass nanopores. (k) Dependence of τ_backward_/τ_forward_ on pore diameter in glass nanopores. (l) 10-s excerpts of raw ionic-current traces for dsDNA in glass (top) and SiN*_x_* (bottom) nanopores. (m) Comparison of translocation frequencies between glass and SiN*_x_* nanopores. The sample concentration is 1.75 nM. Error bars represent SDs from measurements on three independent nanopores. (n) Representative unperturbed events in SiN*_x_* nanopores. (o) Representative perturbed events in SiN*_x_* nanopores. (p) Perturbed-event fractions for dsDNA (Mol A), the DNA construct (Mol B), and λ-DNA (Mol C), respectively. No perturbed events were observed in glass nanopores (0%).

Figure 4b and c present typical scatter plots of maximum current blockade versus dwell time of dsDNA translocation in glass and SiN_*x*_ nanopores, respectively. In both cases, two clusters are evident, corresponding to unfolded (linear) and folded translocation. As illustrated in Figure 4d, folded events arise when an internal DNA segment is captured at the pore mouth; these events complicate signal interpretation and are generally undesirable.^72^ We first compared the folded-event fraction under the operating directions used for each platform: from the tip toward the interior for the conical glass nanopore, and from the thinned membrane toward the Si support (trapezoidal base) for the SiN_*x*_ nanopore. In 4 M LiCl, both platforms yielded folded fractions of ∼75% (Figure 4e), indicating that, under these conditions, the prevalence of folded versus linear translocation is largely platform-independent. We then examined directionality in the conical glass nanopore by reversing the applied bias. In this context, forward translocation refers to motion from the tip toward the interior, while backward translocation denotes the opposite direction (Figure 4f). The folded-event fraction was approximately 75% in the forward direction but decreased to about 45% in the backward direction (Figure 4g). This reduction observed in the backward (interior-to-tip) configuration likely arises from the lower probability of internal loop formation within the confinement.

Direction also affected translocation speed, an asymmetric phenomenon we previously reported and leveraged for molecular “ping-pong”.^41,73^ In SiN_*x*_ nanopores, forward and backward τ_d_ were nearly identical (∼1.1 ms; Figure 4h–i). In glass nanopores, however, backward events were markedly slower (∼4 ms; Figure 4j and Supplementary Figures S15–S16) than forward events (∼1.08 ms), giving a dwell-time ratio τ_backward_/τ_forward_ ∼ 3.7. Across glass nanopores of different diameters, τ_backward_/τ_forward_ increased as diameter decreased (Figure 4k), indicating enhanced directional rectification in narrower tips and demonstrating that the conical geometry can substantially slow biomolecule translocation.

We also compared capture behavior. Figure 4l shows 10-s excerpts of raw current traces for dsDNA in glass (top) and SiN_*x*_ (bottom) nanopores. Translocation frequencies extracted from 30-min recordings (Figure 4m) were 0.79 nM^–1^·s^–1^ for SiN_*x*_ and 0.47 nM^–1^·s^–1^ for glass, consistent with a lower effective free-energy barrier for the shorter SiN_*x*_ nanopore.

Finally, in SiN_*x*_ nanopores, we observed two distinct classes of translocation signals. Representative unperturbed events—clean, well-defined blockades characteristic of complete DNA passage—are shown in Figure 4n. In contrast, we frequently detected perturbed events, which appear as brief, shallow, and noisy blockades preceding the full translocation signal (Figure 4o). We propose that these perturbed events likely stem from transient interactions of the DNA with the exterior pore mouth. The perturbed-event fractions were 33.6%, 36.3%, and 29.8% for dsDNA, the DNA construct, and λ-DNA, respectively (Figure 4p). No perturbed events were observed in glass nanopores (0%), consistent with stronger electrostatic repulsion from the more negatively charged SiO_2_ surface (∼−35 mC·m^–2^)^74^ compared with SiN_*x*_ (∼−20 mC·m^–2^)^75^ at pH 9. See Supplementary Figure S17 for example perturbed events of dsDNA and λ-DNA. These perturbed events introduce a practical disadvantage for quantitative sensing, as identifying and filtering them reduces data yield and complicates analysis. We utilized a pH 9 buffer to reduce DNA sticking without denaturing dsDNA at higher pH.^76^ While we used 4 M LiCl to improve the nanopore’s spatial and temporal resolution,^77^ further actions, such as decreasing salt concentrations to modulate electrostatic screening, may mitigate these transient interactions.

### Comparison of Sensing Proteins

In addition to DNA molecules, proteins represent another major class of biological targets for nanopore sensing. Unlike DNA, which behaves as a long, uniformly charged polymer, proteins exhibit diverse shapes, sizes, and charge distributions. To evaluate protein sensing performance, we investigated four proteins with different molecular weights: Streptavidin (∼52 kDa), Bovine Serum Albumin (BSA, ∼66.5 kDa), Immunoglobulin G (IgG, ∼150 kDa), and an Alpha-fetoprotein dimer (AFP–hFc, ∼210 kDa), using both glass and SiN_*x*_ nanopores with 10 nm diameters. Figure 5a,c show representative ionic-current traces and scatter plots of translocation time (τ_d_) versus current blockade (ΔI) for each protein. The mean current blockade values (Δ̅I), obtained from Gaussian fitting, are summarized in Figure 5e. In line with the DNA measurements, the Δ̅I values obtained in SiN_*x*_ nanopores are consistently larger than those in glass nanopores, yielding mean blockades of 50, 56, 142, and 205 pA for glass, and 265, 252, 707, and 946 pA for SiN_*x*_, corresponding to Streptavidin, BSA, IgG, and AFP, respectively. The Δ̅I values exhibit a strong linear correlation with protein molecular weight for both nanopore types (Figure 5f). The corresponding SNRs for glass nanopores are 7.9, 8.7, 22.3, and 32.2, while those for SiN_*x*_ nanopores are 9.9, 9.5, 26.5, and 35.4, indicating consistently higher SNRs in SiN_*x*_ nanopores. The average SNR ratio of SiN_*x*_ to glass nanopores is ∼1.16 at a 10 nm scale.

**Figure 5:**
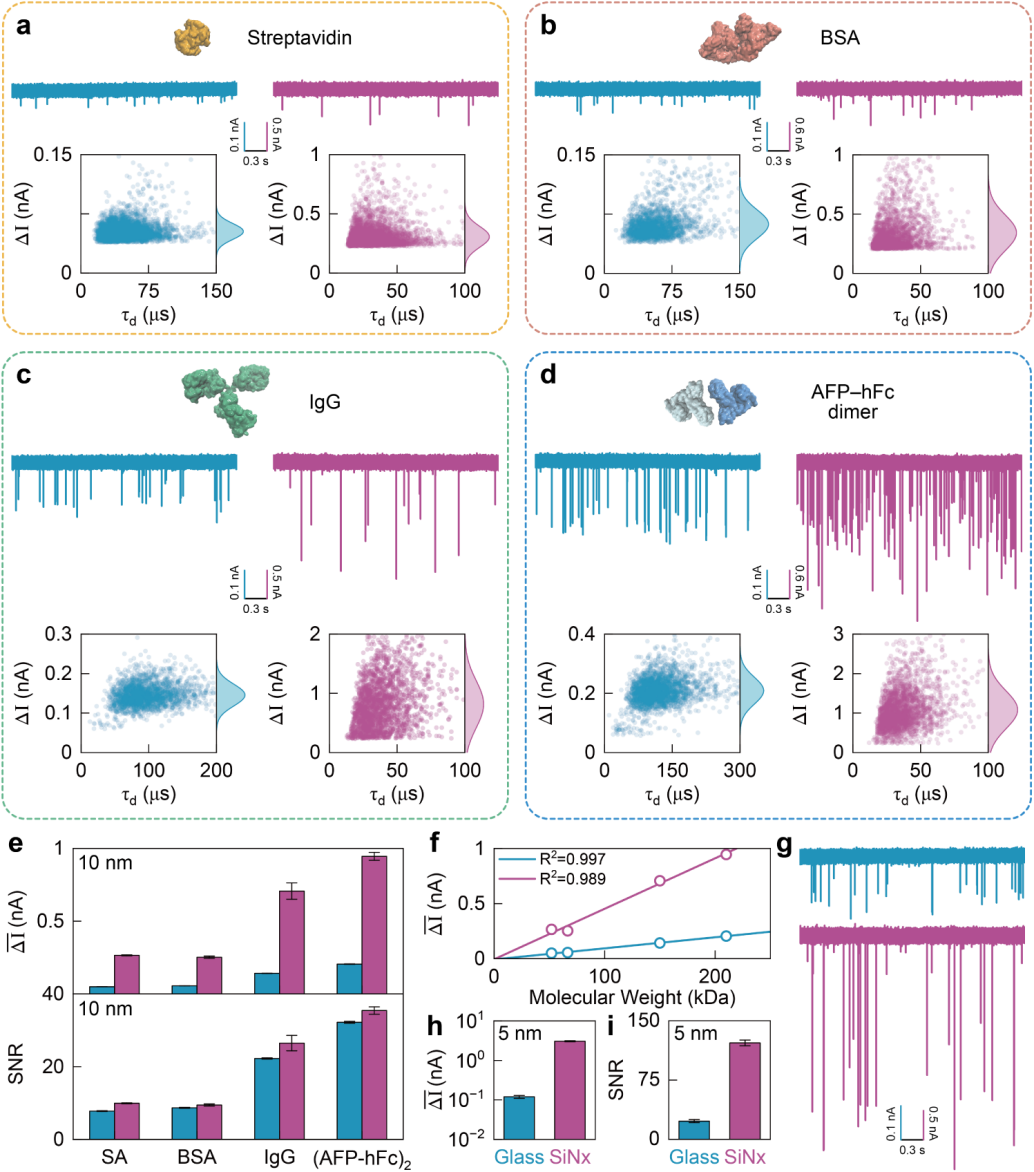
Comparative protein sensing performance of glass and SiN*_x_* nanopores. (a–d) Representative ionic-current traces and scatter plots of translocation time (τ_d_) versus current blockade (ΔI) for streptavidin, BSA, IgG, and AFP–hFc dimer measured using 10 nm glass (blue) and SiN*_x_* (magenta) nanopores. (e) Mean current blockade values (Δ̅I, top) and corresponding SNRs (bottom) for the four proteins on both nanopore platforms. The error bars represent SDs obtained from the Gaussian fitting of the current blockade distribution. (f) Linear dependence of Δ̅I on protein molecular weight for both glass and SiN*_x_* nanopores. (g) Representative current traces of BSA translocation through 5 nm glass (top) and SiN*_x_* (bottom) nanopores. (h) Comparison of Δ̅I values between glass and SiN*_x_* for BSA measured with 5 nm nanopores. (i) Corresponding SNR values for a 5 nm glass and SiN*_x_* nanopore.

To further assess performance at smaller pore sizes, we employed 5 nm nanopores for comparative BSA sensing, as BSA’s effective diameter (∼4 nm) closely matches this pore size. This specific size-matching maximizes the volume exclusion effect, providing an ideal comparison for how each platform handles compact biomolecules approaching the pore’s physical limit. Figure 5g displays representative ionic current traces for BSA translocation events through glass (top) and SiN_*x*_ (bottom) nanopores. The Δ̅I values increase significantly as the nanopore diameter decreases, from 0.056 nA to 0.12 nA for glass and from 0.25 nA to 3.0 nA for SiN_*x*_ (Figure 5h and Supplementary Figure S18). Correspondingly, the SNR values increase to ∼22 for glass and dramatically to ∼121 for SiN_*x*_, with the latter exhibiting approximately 5-fold higher SNR than glass nanopores of the same diameter. See Supplementary Figure S23 for the SNR of a medium nanopore size (∼7 nm) for both platforms.

We summarize the sensing performance of glass and SiN_*x*_ nanopores in Figure 6. At a 10 nm diameter, glass and SiN_*x*_ nanopores exhibit comparable performance for both DNA and proteins (Figure 6a–b). When the diameter is reduced to 5 nm, closer to the size of typical target biomolecules, the trends diverge: glass nanopores provide stronger DNA sensing (Supplementary Figure S19, SNR > 80), whereas SiN_*x*_ nanopores deliver higher protein sensitivity (Figure 6b, SNR > 120). These observations suggest a practical detection guideline: all else being equal, longer nanopores tend to favor long polymers, whereas shorter nanopores favor small molecules.

**Figure 6:**
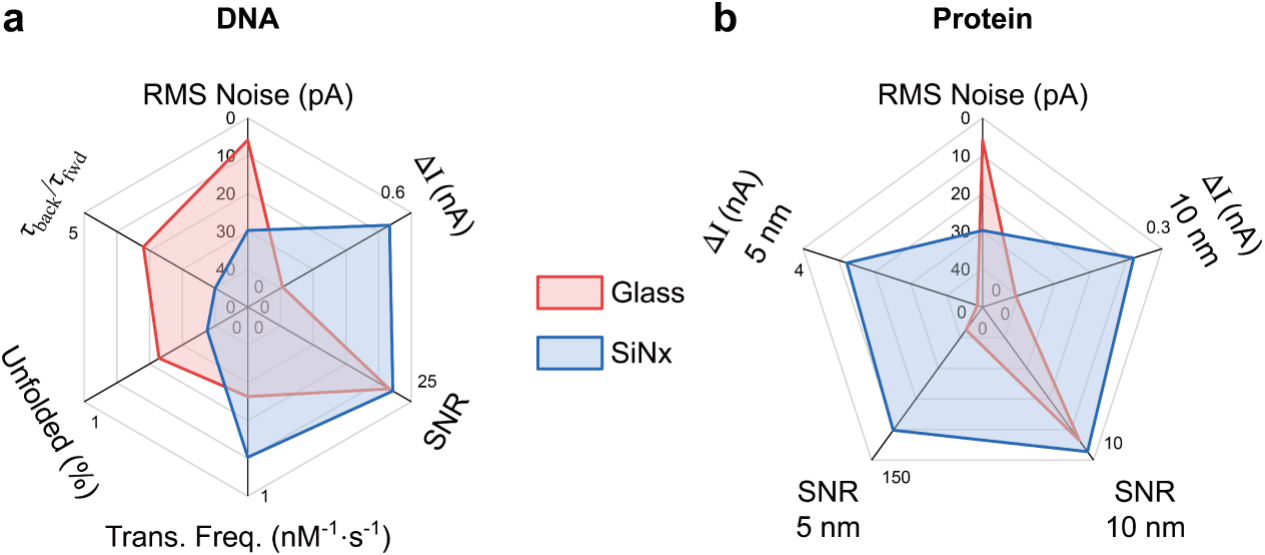
Comparison of sensing performance between glass and SiN*_x_* nanopores for DNA and protein detection. (a) Spider plots summarizing key performance metrics for DNA sensing, including RMS noise, current blockade (ΔI), SNR, translocation frequency, and unfolded event ratio. These performance metrics were measured in 10 nm nanopores. (b) Comparison for protein detection. Both ΔI and SNRs of 10 nm and 5 nm glass and SiN*_x_* nanopores are listed. The comparison of DNA sensing performance between the two platforms in different nanopore sizes is provided in Supplementary Figure S19.

The mechanisms, however, are not explained by pore length alone. For DNA, the field strength is expected to increase when a DNA strand occupies the nanopore.^14^ In our simulation, the electric field at a 5 nm glass nanopore is approximately 2.1× that of a 10 nm pore (Supplementary Figure S14), compared with 1.17× in SiN_*x*_ nanopores. The greater increment of the electric field in the glass nanopore when decreasing nanopore size suggests that it will yield higher current density and, thus, higher current blockade in the signal.

For proteins, the higher SNR in SiN_*x*_ nanopores is consistent with a volume-exclusion mechanism.^78^ Proteins typically reside fully inside the pore, while only a fraction of a DNA molecule is confined at any instant. Because SiN_*x*_ pores are short and have a much smaller effective pore volume than tapered glass nanopores (whose effective length is on the order of hundreds of nanometers; Supporting Information Figures S5 and S14), the fractional volume exclusion, and thus the current blockade, is larger in SiN_*x*_, yielding superior protein sensitivity.

Geometry also affects other performance metrics. The conical shape of glass nanopores enables a backward translocation configuration that extends DNA dwell times by ∼3–7× and substantially reduces the fraction of folded events. However, these benefits come at the cost of lower capture rates compared with short SiN_*x*_ nanopores. Material properties further differentiate the platforms. Owing to the lower dielectric permittivity of SiO_2_, glass nanopores exhibit substantially reduced capacitive noise, leading to ∼4–5× lower RMS current noise under identical conditions.

Apart from sensing performance, the usability of a nanosensor also matters.^79,80^ We compare that of glass and SiN_*x*_ nanopore platforms in Supporting Information Table S6. Glass nanopores are simpler to fabricate, whereas SiN_*x*_ nanopores require specialized instrumentation and expertise. Both platforms exhibit high repeatability for nanopores of >5 nm. Glass nanopores offer faster fabrication, though creating those of <5 nm presents challenges. Both types are reusable with proper storage. In terms of access to samples in the *trans* reservoir, glass nanopores are more restricted due to their narrow geometry, which complicates buffer exchange, sample handling, and the analysis of translocated biomolecules. By contrast, SiN_*x*_ nanopore chips typically provide straightforward access to both the *cis* and *trans* reservoirs, allowing more flexible experimental configurations. While both are mechanically stable, the capillary tips of glass nanopores and the SiN_*x*_ membrane are prone to breakage during handling.

We summarize previously reported SNR values for glass and SiN_*x*_ nanopores from the literature in Supporting Information Table S4 for comparison. It is important to note that SNR is influenced by multiple experimental parameters, including the filter cutoff frequency (which affects RMS noise), nanopore size, applied voltage, and analyte dimensions, which determine the magnitude of current blockade. Consequently, a fully parameter-normalized comparison with the prior literature is not straightforward. From another angle, this difficulty motivates our head-to-head evaluation of glass and SiN_*x*_ nanopores under the same experimental conditions.

Although our results suggest a general guideline that glass nanopores are better suited for DNA sensing, whereas SiN_*x*_ nanopores perform better for proteins, this criterion is not universal. For instance, if a protein is unfolded into an extended polymer chain, glass nanopores may offer higher sensitivity due to their geometry and field focusing. Conversely, for short oligonucleotides, the smaller sensing volume of SiN_*x*_ nanopores can provide superior detection performance.

## Conclusions

In sum, this study provides a quantitative, mechanistic basis for choosing between glass and SiN_*x*_ nanopores, delivering a decision framework that links architecture to the analyte class, measurement priorities, and platform-level limitations. We anticipate that these insights will guide both sensor selection in current applications and the design of next-generation nanopore instruments.

## Methods

### Nanopore Fabrication

Silicon nitride nanopore chips were fabricated by using MEMS technologies. A SiN_*x*_ layer was deposited via chemical vapor deposition, followed by photolithographic patterning, localized membrane thinning, and silicon etching to release the thin, free-standing membranes. SiN_*x*_ nanopores were fabricated by TEM drilling of the membrane. Glass nanopores were fabricated by pulling quartz glass capillaries by using a laser puller to achieve conical geometries with nanoscale tip diameters. The pulled glass nanopores were subsequently integrated into custom microfluidic chips. Both nanopore platforms underwent plasma cleaning and hydrophilic surface treatments prior to use to ensure wettability and stable baseline current. Pore size characterization was performed by using TEM.

### Nanopore Measurement

Single-molecule translocation experiments and electrical measurements were performed using a high-bandwidth patch-clamp amplifier. Ag/AgCl electrodes were immersed in the *cis* and *trans* reservoirs to apply a voltage across the nanopore and measure the resulting ionic current. Current–voltage characteristics were recorded to characterize pore size. Ionic current signals were sampled at 1 MHz and low-pass filtered at 50 kHz. Data acquisition and extraction of translocation dynamics, including current blockade, dwell time, and signal-to-noise ratio, were performed using custom-written Python scripts.

### Finite Element Analysis

Finite-element analysis was performed using COMSOL Multiphysics. A 2D axisymmetric model was constructed to simulate the geometries of the SiN_*x*_ nanopores and the conical glass nanopore. By coupling the Poisson–Nernst–Planck (PNP) equations for ion transport with the Navier–Stokes equations for fluid dynamics, the spatial distribution of the electric field across the pores was simulated.

## Supplementary Material



## Notes

MEMS fabrication process for SiN_*x*_ nanopore chip wafers; fabrication of glass nanopores and SiN_*x*_ nanopores; nanopore measurements; preparation of DNA samples; materials used in experiments; finite element method for analyzing the two nanopores; and supplementary figures (PDF)

## References

[1] Kasianowicz J. J., Brandin E., Branton D. (1996). Characterization of
Individual Polynucleotide Molecules Using a Membrane Channel. Proc. Natl. Acad. Sci. U. S. A..

[2] Xue L., Yamazaki H., Ren R. (2020). Solid-State Nanopore
Sensors. Nat. Rev. Mater..

[3] Fried J. P., Swett J. L., Nadappuram B. P. (2021). In Situ Solid-State
Nanopore Fabrication. Chem. Soc. Rev..

[4] Li J., Stein D., McMullan C. (2001). Ion-Beam Sculpting at
Nanometre Length Scales. Nature.

[5] Storm A. J., Chen J. H., Ling X. S. (2003). Fabrication of Solid-State
Nanopores with Single-Nanometre Precision. Nat.
Mater..

[6] Steinbock L. J., Otto O., Chimerel C. (2010). Detecting DNA Folding
with Nanocapillaries. Nano Lett..

[7] Guan X., Li H., Chen L. (2023). Glass Capillary-Based Nanopores for Single
Molecule/Single Cell Detection. ACS Sens..

[8] Horne R. I., Sandler S. E., Vendruscolo M. (2025). Detection of Protein
Oligomers with Nanopores. Nat. Rev. Chem..

[9] Dekker C. (2007). Solid-State
Nanopores. Nat. Nanotechnol..

[10] Venkatesan B. M., Bashir R. (2011). Nanopore Sensors for
Nucleic Acid Analysis. Nat. Nanotechnol..

[11] Miles B. N., Ivanov A. P., Wilson K. A. (2013). Single Molecule Sensing
with Solid-State Nanopores: Novel Materials, Methods, and Applications. Chem. Soc. Rev.

[12] Zheng F., Suma A., Maffeo C., Chen K., Alawami M., Sha J., Aksimentiev A., Micheletti C., Keyser U. F. (2025). Torsion-Driven Plectoneme
Formation During Nanopore Translocation of DNA Polymers. Phys. Rev. X.

[13] Chen K., Jou I., Ermann N. (2021). Dynamics of Driven Polymer Transport through
a Nanopore. Nat. Phys..

[14] Chen K., Choudhary A., Sandler S. E., Maffeo C., Ducati C., Aksimentiev A., Keyser U. F. (2023). Super-Resolution Detection of DNA
Nanostructures Using a Nanopore. Adv. Mater..

[15] Lee Y., Muthukumar M. (2025). Charge Symmetry Breaking in Neutral Polyzwitterions. Nat. Commun..

[16] Chou Y.-C., Lin C.-Y., Castan A. (2024). Coupled Nanopores for
Single-Molecule Detection. Nat. Nanotechnol..

[17] Sandler S. E., Horne R. I., Rocchetti S. (2023). Multiplexed Digital
Characterization of Misfolded Protein Oligomers via Solid-State Nanopores. J. Am. Chem. Soc..

[18] Soni N., Rosenstock Z., Verma N. C., Siddharth K., Talor N., Liu J., Marom B., Kolomeisky A. B., Aksimentiev A., Meller A. (2025). Full-Length Protein Classification
via Cysteine Fingerprinting in Solid-State Nanopores. Nat. Nanotechnol..

[19] Schmid S., Stömmer P., Dietz H. (2021). Nanopore Electro-Osmotic
Trap for the Label-Free Study of Single Proteins and Their Conformations. Nat. Nanotechnol..

[20] Wang X., Thomas T.-M., Ren R. (2023). Nanopore Detection Using
Supercharged Polypeptide Molecular Carriers. J. Am. Chem. Soc..

[21] Tripathi P., Benabbas A., Mehrafrooz B., Yamazaki H., Aksimentiev A., Champion P. M., Wanunu M. (2021). Electrical
Unfolding of Cytochrome
c during Translocation through a Nanopore Constriction. Proc. Natl. Acad. Sci. U. S. A..

[22] Confederat S., Lee S., Vang D., Soulias D., Marcuccio F., Peace T. I., Edwards M. A., Strobbia P., Samanta D., Wälti C. (2024). Next-Generation Nanopore
Sensors Based on Conductive
Pulse Sensing for Enhanced Detection of Nanoparticles. Small.

[23] Huang B., Miao L., Li J., Xie Z., Wang Y., Chai J., Zhai Y. (2022). Identification of Plasmon-Driven
Nanoparticle-Coalescence-Dominated Growth of Gold Nanoplates through
Nanopore Sensing. Nat. Commun..

[24] Kawaguchi T., Tsutsui M., Murayama S., Leong I. W., Yokota K., Komoto Y., Taniguchi M. (2024). Enhanced Nanoparticle
Sensing in
a Highly Viscous Nanopore. Small Methods.

[25] Ren R., Sun M., Goel P., Cai S., Kotov N. A., Kuang H., Xu C., Ivanov A. P., Edel J. B. (2021). Single-Molecule
Binding Assay Using
Nanopores and Dimeric NP Conjugates. Adv. Mater..

[26] Tsutsui M., Wada M., Arima A. (2024). Identifying
Viral Vector
Characteristics by Nanopore Sensing. ACS Nano.

[27] Leach, A. R. Nanopipettes as a Tool to Study Single Biological Entities; University of Leeds, 2022.

[28] Bandara Y. M. N. D. Y., Freedman K. J. (2022). Enhanced Signal to Noise Ratio Enables
High Bandwidth
Nanopore Recordings and Molecular Weight Profiling of Proteins. ACS Nano.

[29] Rosenstein J. K., Wanunu M., Merchant C. A. (2012). Integrated Nanopore
Sensing Platform with Sub-Microsecond Temporal Resolution. Nat. Methods.

[30] Chien C.-C., Shekar S., Niedzwiecki D. J. (2019). Single-Stranded DNA
Translocation Recordings through Solid-State Nanopores on Glass Chips
at 10 MHz Measurement Bandwidth. ACS Nano.

[31] Lin C.-Y., Fotis R., Xia Z. (2022). Ultrafast Polymer Dynamics
through a Nanopore. Nano Lett..

[32] Fragasso A., Schmid S., Dekker C. (2020). Comparing Current Noise in Biological
and Solid-State Nanopores. ACS Nano.

[33] Leitao S. M., Navikas V., Miljkovic H. (2023). Spatially Multiplexed
Single-Molecule Translocations through a Nanopore at Controlled Speeds. Nat. Nanotechnol..

[34] Liu W., Zhang Y., Gu Z. (2024). Structure Design of
Silicon-Based Nanopore Chips for Noise Reduction. IEEE Sens. J..

[35] Chau C., Marcuccio F., Soulias D. (2022). Probing RNA Conformations
Using a Polymer–Electrolyte Solid-State Nanopore. ACS Nano.

[36] Shi X., Pumm A.-K., Maffeo C. (2024). A DNA Turbine Powered
by a Transmembrane Potential across a Nanopore. Nat. Nanotechnol..

[37] Yazbeck R., Xu Y., Porter T., Duan C. (2022). Nanoparticle-Blockage-Enabled
Rapid
and Reversible Nanopore Gating with Tunable Memory. Proc. Natl. Acad. Sci. U. S. A..

[38] Pandey L., Panigaj M., Radwan Y. (2025). Chemical
Composition
and Backbone Modifications Define Deformability of Nucleic Acid Nanoparticles. ACS Nano.

[39] Chau C. C., Maffeo C. M., Aksimentiev A., Radford S. E., Hewitt E. W., Actis P. (2024). Single
Molecule Delivery into Living Cells. Nat. Commun..

[40] Sandler S. E., Weckman N. E., Yorke S. (2024). Sensing the DNA-Mismatch
Tolerance of Catalytically Inactive Cas9 via Barcoded DNA Nanostructures
in Solid-State Nanopores. Nat. Biomed. Eng..

[41] Zheng F., Alawami M., Zhu J. (2023). DNA Carrier-Assisted
Molecular Ping-Pong in an Asymmetric Nanopore. Nano Lett..

[42] Lastra L. S., Bandara Y. M. N. D. Y., Nguyen M., Farajpour N., Freedman K. J. (2022). On the Origins of Conductive Pulse Sensing inside a
Nanopore. Nat. Commun..

[43] Zheng F., Li H., Yang J. (2024). Modulation
of Ion Transport in Nanopores Using
Polyethylene Glycol. Langmuir.

[44] Kwon S.-R., Fu K., Han D. (2018). Redox Cycling in Individually Encapsulated
Attoliter-Volume Nanopores. ACS Nano.

[45] Hu P., Wang Y., Zhang Y. (2022). Glass Nanopore Detection
of Copper Ions in Single Cells Based on Click Chemistry. Anal. Chem..

[46] Liu Y.-L., Yu S.-Y., An R. (2023). A Fast and Reversible
Responsive Bionic Transmembrane Nanochannel for Dynamic Single-Cell
Quantification of Glutathione. ACS Nano.

[47] Wang B., Xu Y.-T., Zhang T.-Y. (2024). An Ultrasensitive and
Efficient microRNA Nanosensor Empowered by the CRISPR/Cas Confined
in a Nanopore. Nano Lett..

[48] Su X., Yusuf M. L., Guo X. (2025). Recent Advances of Nucleic
Acids-Based Nanopipette: From Fundamental to Applications. Anal. Chem..

[49] Fu K., Bohn P. W. (2018). Nanopore Electrochemistry:
A Nexus for Molecular Control
of Electron Transfer Reactions. ACS Cent. Sci..

[50] Wang Y., Yang H., Li Z. (2025). Programming Ion Gating
and Dynamic Response of Nanopores with Responsive Tetrahedral DNA
Nanostructures. Anal. Chem..

[51] Ahmed S. A., Liu Y., Xiong T. (2024). Iontronic Sensing Based on Confined Ion Transport. Anal. Chem..

[52] Zhu C., Huang K., Siepser N. P. (2021). Scanning Ion Conductance
Microscopy. Chem. Rev..

[53] Sun L., Shigyou K., Ando T. (2019). Thermally Driven Approach
To Fill Sub-10-Nm Pipettes with Batch Production. Anal. Chem..

[54] Cadinu P., Kang M., Nadappuram B. P. (2020). Individually Addressable
Multi-Nanopores for Single-Molecule Targeted Operations. Nano Lett..

[55] Cadinu P., Paulose Nadappuram B., Lee D. J. (2017). Single Molecule Trapping
and Sensing Using Dual Nanopores Separated by a Zeptoliter Nanobridge. Nano Lett..

[56] Chou Y.-C., Masih Das P., Monos D. S. (2020). Lifetime and Stability
of Silicon Nitride Nanopores and Nanopore Arrays for Ionic Measurements. ACS Nano.

[57] Waugh M., Briggs K., Gunn D. (2020). Solid-State Nanopore
Fabrication by Automated Controlled Breakdown. Nat. Protoc..

[58] Ivanov A. P., Instuli E., McGilvery C. M. (2011). DNA Tunneling Detector
Embedded in a Nanopore. Nano Lett..

[59] Fanget A., Traversi F., Khlybov S. (2014). Nanopore Integrated
Nanogaps for DNA Detection. Nano Lett..

[60] Xie P., Xiong Q., Fang Y. (2012). Local Electrical Potential
Detection of DNA by Nanowire–Nanopore Sensors. Nat. Nanotechnol..

[61] Tsutsui M., Hsu W.-L., Hsu C., Garoli D., Weng S., Daiguji H., Kawai T. (2025). Transmembrane
Voltage-Gated Nanopores
Controlled by Electrically Tunable in-Pore Chemistry. Nat. Commun..

[62] Jain T., Rasera B. C., Guerrero R. J. S. (2017). Microfluidic Multiplexing
of Solid-State Nanopores. J. Phys.: Condens.
Matter.

[63] Ivankin A., Henley R. Y., Larkin J. (2014). Label-Free Optical Detection
of Biomolecular Translocation through Nanopore Arrays. ACS Nano.

[64] Hu R., Zhu R., Wei G., Wang Z., Gu Z.-Y., Wanunu M., Zhao Q. (2023). Solid-State Quad-Nanopore Array for High-Resolution
Single-Molecule Analysis and Discrimination. Adv. Mater..

[65] He Y., Tsutsui M., Zhou Y., Miao X.-S. (2021). Solid-State Nanopore
Systems: From Materials to Applications. NPG
Asia Mater..

[66] Lee K., Park K.-B., Kim H.-J., Yu J.-S., Chae H., Kim H.-M., Kim K.-B. (2018). Recent Progress in Solid-State
Nanopores. Adv. Mater..

[67] van
den Hout M., Hall A. R., Wu M. Y. (2010). Controlling
Nanopore Size, Shape and Stability. Nanotechnology.

[68] Lin K., Chen C., Li D. (2025). Capacitance Effects
of Nanopore Chips on Ionic Current Modulation and Noise Characteristics. Nanotechnology.

[69] Tabard-Cossa V., Trivedi D., Wiggin M. (2007). Noise
Analysis and Reduction
in Solid-State Nanopores. Nanotechnology.

[70] Bell N. A. W., Keyser U. F. (2016). Digitally Encoded
DNA Nanostructures for Multiplexed,
Single-Molecule Protein Sensing with Nanopores. Nat. Nanotechnol..

[71] Alawami M. F., Bošković F., Zhu J. (2022). Lifetime
of Glass Nanopores in a PDMS Chip for Single-Molecule Sensing. iScience.

[72] Schmidt T. T., Earle M. K., Patiño-Guillén G., Li Y., Alexii R.-E., Baumberg J. J., Keyser U. F., Platnich C. M. (2025). Supramolecular
Interactions Modulate RNA: DNA Folding Observed via Nanopore Sensing. Angew. Chem., Int. Ed..

[73] Bell N. A. W., Chen K., Ghosal S., Ricci M., Keyser U. F. (2017). Asymmetric Dynamics of DNA Entering and Exiting a Strongly Confining
Nanopore. Nat. Commun..

[74] Behrens S. H., Grier D. G. (2001). The Charge of Glass and Silica Surfaces. J. Chem. Phys..

[75] Lin K., Li Z., Tao Y. (2021). Surface Charge Density Inside a Silicon Nitride
Nanopore. Langmuir.

[76] Fologea D., Gershow M., Ledden B. (2005). Detecting Single Stranded
DNA with a Solid State Nanopore. Nano Lett..

[77] Kowalczyk S. W., Wells D. B., Aksimentiev A. (2012). Slowing down DNA Translocation
through a Nanopore in Lithium Chloride. Nano
Lett..

[78] Li M.-Y., Ying Y.-L., Yu J. (2021). Revisiting the Origin
of Nanopore Current Blockage for Volume Difference Sensing at the
Atomic Level. JACS Au.

[79] Chen Y., Fu K. X., Cotton R. (2025). A Biochemical Sensor
with Continuous Extended Stability in Vivo. Nat. Biomed. Eng..

[80] Seo J.-W., Fu K., Correa S., Eisenstein M., Appel E. A., Soh H. T. (2022). Real-Time
Monitoring of Drug Pharmacokinetics within Tumor Tissue
in Live Animals. Sci. Adv..

