# Supplementary material for: Comparative Benchmarking of Glass and Silicon Nitride Nanopores for Single-Molecule Detection
Source: ACS Nano. 2026 May 5;20(19):14256–67. doi: 10.1021/acsnano.6c03089 (PMC13192321; doi:10.1021/acsnano.6c03089)
Supplement: Supplementary file 1 [file nn6c03089_si_001.pdf]

Supplementary Materials for

Comparative Benchmarking of Glass and Silicon Nitride Nanopores for Single-Molecule Detection

Fei Zheng[1,2#*], Zhan Wang[3#], An Bai[4], Rui Hu[1], Xianhu Sun[4], Jingjie Sha[5], Qing Zhao[3], Kaikai Chen[1*], Ulrich F. Keyser[2*]

1. School of Nanoscience and Nanotechnology, University of Chinese Academy of Sciences, Beijing 101408, China

2. Cavendish Laboratory, University of Cambridge, Cambridge CB3 0US, United Kingdom

3. State Key Lab for Mesoscopic Physics and Frontiers Science Center for Nano-optoelectronics, Electron Microscopy Laboratory, School of Physics, Peking University, Beijing 100871, China

4. School of Chemical Sciences, University of Chinese Academy of Sciences, Beijing 101408, China

5. Jiangsu Key Laboratory for Design and Manufacture of Precision Medicine Equipment, School of Mechanical Engineering, Southeast University, Nanjing 211189, China

[*]Correspondence to: fz284@cam.ac.uk; chenkaikai@ucas.ac.cn; ufk20@cam.ac.uk

This PDF file includes:

Supplementary Notes 1 – 7

Figure S1 to S23

Table S1 to S6

Supplementary Notes

1. MEMS Fabrication Process for SiN_x Nanopore Chip Wafers

Layer Deposition

Fabrication began with a 220 μm-thick silicon wafer subjected to sequential piranha cleaning (H_2SO_4:H_2O_2 = 3:1) and standard RCA steps to remove organic and ionic contaminants. A 1-μm SiO_2 layer was then thermally grown at 1000 °C in dry O_2 to serve as an etch stop. Subsequently, a 100-nm Si_3N_4 film was deposited by low-pressure chemical vapor deposition (LPCVD) at 750 °C using dichlorosilane (SiH_2Cl_2) and ammonia (NH_3) precursors. Film stress was managed to limit wafer bow to < 5 μm.

$$3SiH_2Cl_2+4NH_3 \xrightarrow{750^{\circ}C,30Pa} Si_3N_4+Gas \tag{S1}$$

Front-Side Patterning and Membrane Thinning

The front-side Si_3N_4 layer was patterned by spin-coating LC100A photoresist at 2000 rpm, followed by UV exposure through a chrome mask defining 2.5 μm circular features. After development in MF-319, reactive ion etching (RIE) in a CHF_3/O_2 plasma (20 sccm, 100 W RF power) thinned the exposed Si_3N_4 from 100 nm to 15 nm, forming a locally thinned region for nanopore placement. Residual resist was removed by oxygen plasma ashing and acetone rinsing.

Back-Side Etch Window Formation

Using alignment to the front-side features with < 1 μm tolerance, a 323 μm × 323 μm back-side etch window was defined by photolithography. A two-step RIE sequence first cleared the back-side Si_3N_4, then etched the underlying SiO_2 to expose the silicon substrate.

Bulk Silicon Etching and Membrane Release

Bulk silicon was anisotropically etched in 30 wt% KOH at 50 °C at an average rate of 11 $\mu m \cdot h^{-1}$, terminating on the SiO_2 etch-stop after approximately 20 h. The resulting cavity exhibited {111} sidewalls with 54.7° facets. The wafer was then immersed in buffered oxide etch (BOE, 6:1 NH_4F:HF) for 13 min to dissolve the 1-μm SiO_2 layer, releasing a 20 × 20 μm^2 free-standing Si_3N_4 membrane.

Figure S1 summarizes the MEMS fabrication workflow, and Figure S2 details the SiN_x membrane chip architecture. The 2.5 μm circular region is locally thinned by approximately 85 nm (from 100 nm to 15 nm), as characterized previously by AFM scanning[1]).

2. Fabrication of Glass Nanopores

Quartz glass capillaries (inner diameter 0.2 mm, outer diameter 0.5 mm) were purchased from Sutter Instrument (California, USA). The capillaries were then pulled on a laser-assisted micropipette puller (P-2000/F, Sutter Instrument, California, USA) to achieve target nanopipette tip diameters of 10 nm and 5 nm, with a standard deviation of ± 2 nm. The specific pulling parameters used in this work are provided in Supplementary Table S1; additional guidance on parameter selection can be found in the P-2000 user guide. After pulling, each capillary was cut to the desired length and positioned in the reservoir of a custom polydimethylsiloxane (PDMS) microfluidic chip fabricated from the Sylgard 184 silicone elastomer kit (The Dow Chemical Company, MI, USA). Following the same procedure, eight capillaries were assembled on a single chip, shown in Supplementary Figure S20. Prior to bonding, the PDMS device and a glass slide were activated by oxygen plasma (Femto, Diener Electronic, Germany) at a chamber pressure of 0.25 mbar with an O_2 flow of 14 sccm for 20 s and then brought into conformal contact. Liquid PDMS prepolymer was applied around the capillaries to seal the interfaces and cured at 140 °C for 30 min. The assembled chip was subsequently treated with oxygen plasma again for 20 min to render the nanopore surfaces hydrophilic. Finally, the chambers were filled with 4 M LiCl in 1× TE buffer (pH 9.0) to wet and prime the nanopores. Two Ag/AgCl electrodes were oxidized by immersing Ag wires into NaClO solution (10%), and then inserted into the central reservoir (*cis*) and the outer reservoir (*trans*), respectively, to establish an electric circuit across the nanopore. The openings of each chamber were sealed using a tape to avoid water evaporation which will lead to the LiCl concentration increase in solution. Before sample measurements, the current-voltage characteristic of nanopores was scanned from -600 mV to 600 mV to calculate the pore diameter. All experiments were performed with a root-mean-square (RMS) noise below 7.0 pA for the baseline current.

3. Fabrication of SiN_x Nanopores

Low-stress SiN_x membrane chips (window size 20 × 20 μm^2, membrane thickness 15 nm) were fabricated as described in Supplementary Note 1. The chips were sequentially rinsed with acetone, isopropanol, and deionized water (Milli-Q, Merck Millipore, Darmstadt, Germany), dried with filtered N_2, and treated with oxygen plasma (Femto, Diener Electronic, Ebhausen, Germany) for 60 s to remove residual hydrocarbons. Nanopores were drilled using a transmission electron microscope (FEI Tecnai F30, Thermo Fisher Scientific, Hillsboro, OR, USA) operated at 300 kV with a focused electron beam. The beam size was set to 5 nm,

corresponding to the full width at half-maximum (FWHM) of the Gaussian intensity profile in the transverse focal plane. This beam size has been reported to produce nanopores with a near-cylindrical geometry[2], minimizing the hourglass shape typical of larger beam conditions. Following drilling, each single-nanopore chip was immersed in piranha solution (3:1 H_2SO_4/H_2O_2) at 90 °C for 5 min to remove organic residues, rinsed three times with deionized water, and stored in deionized water to maintain hydrophilicity. For measurements, each chip was mounted between two elastomer gaskets attached to a custom polymethylmethacrylate (PMMA) fluidic cell, shown in Supplementary Figure S20. Ag/AgCl electrodes were placed in the *cis* and *trans* chambers, prepared by oxidizing silver wires in a 10% NaClO solution. The reservoirs were then filled with 4 M LiCl in 1× TE buffer (pH 9.0) for ionic current measurements.

4. Nanopore Measurements

TEM imaging was performed in scanning transmission electron microscopy (STEM) mode. For the SiN_x nanopores, characterization was carried out immediately following the electron-beam fabrication process. For the glass nanopores, the pulled quartz capillaries were trimmed, and the resulting tips were affixed to a copper grid (EMCN, Catalog No. AZS205) prior to being loaded into the TEM. All images were processed and analyzed using DigitalMicrograph software to measure the pore diameters. Nanopore measurements were performed using a patch-clamp amplifier (Axopatch 200B, Molecular Devices, CA, USA). Ionic current signals were sampled at 1 MHz and digitized via a data acquisition card (PCI-6251, National Instruments). The current trace was low-pass filtered at 50 kHz using an external Bessel filter (Model 900CT, Frequency Devices), while the applied voltage signal was filtered at 5 kHz with the same model. Data acquisition and real-time monitoring were implemented using a custom LabVIEW-based graphical user interface (National Instruments), and all subsequent analyses were carried out with in-house Python scripts. Translocation events were automatically detected, and when a molecule got stuck within the pore, an automated "kick-out" protocol was triggered by reversing the voltage polarity to remove the blockage.

5. Preparation of DNA Samples

<u>Double-stranded M13mp18 DNA</u>

Double-stranded M13mp18 DNA was enzymatically cut from M13mp18 vectors (M13mp18 RF I DNA, 40 µL, 100 µg/ml, Catalog number N4018S, New England Biolabs (NEB), Hitchin, UK) by mixing it with BamHI-HF (1 µL, 100,000 units/ml, Catalog number R3136M, NEB) and EcoRI-HF (1 µL, 100,000 units/ml, Catalog number R3101M, NEB). Then the mixture was incubated at 37°C for 1 hour and purified using a Monarch PCR & DNA Cleanup Kit (5 µg, Catalog number T1030S, NEB). Enzymes were maintained on ice throughout the entire preparation process. The concentration of final DNA sample was measured using a NanoDrop Spectrophotometer (Nanodrop™ 2000, Thermo Scientific, Waltham, USA).

Lambda DNA

Lambda DNA was purchased from NEB (500 µg/ml, Catalog number N3011S). Because Lambda DNA has two sticky ends with complementary sequences, the samples were heated at 70°C for 10 mins to avoid its self-circularization before measurements. In all processes, the lambda DNA samples were pipetted carefully with large-diameter pipette tips (RNase-free Tips, 200 µL size, Catalog number AM12650, Thermal Fisher Scientific, Waltham, USA) and no vigorous centrifugation to avoid fragmentation.

DNA construct

DNA constructs with six barcoded markers were synthesized as follows.

First is the synthesis of a linear single-stranded M13mp18 scaffold. A 39-base oligonucleotide (5'-TCTAGAGGATCCCCGGGTACCGAGCTCGAATTCGTAATC-3', 2 µL, 100 µM, Integrated DNA Technologies (IDT), Coralville, USA) was hybridized to the M13mp18 scaffold by mixing it with single-stranded m13mp18 circular DNA (40 µL, 250 ng/µL, NEB), 10X rCutSmart Buffer (8 µL, NEB), and DEPC-treated nuclease-free water (28 µL, Ambion, catalog number AM9937). The reaction components were mixed by pipetting and spin down for a couple of seconds, followed by heating to 65°C and linearly cooling to 25°C in a thermocycler (ProFlex™ PCR System, Applied Biosystems™, Foster City, USA) over 40 minutes. The cooling process consisted of 90 cycles of 30 s where temperature decreases by 0.5 °C in each cycle. Then BamHI-HF (1 µL) and EcoRI-HF (1 µL) were then added to the mixture followed by incubation at 37°C for 1 hour. The cut m13mp18 DNA was then immediately purified using the same DNA cleanup kit. The concentration was measured by a NanoDrop Spectrophotometer for the following mixing steps, usually at ~120 nM.

Second is the assembly of the DNA construct. The cut single-stranded linear m13mp18 DNA (8 µL, 100 nM) was mixed with oligonucleotide staples (20 µL in total, each oligo 200 nM, custom oligo pool, IDT), $MgCl_2$ (4 µL, 100 mM), 1.2 µL Tris-EDTA buffer (mixture of

100 mM Tris-HCl (pH=8) and 10 mM EDTA), and 6.8 μL nuclease-free water. The sequences of complementary oligonucleotide staples and oligonucleotides of six markers to form dumbbell structures are shown in Table S2 and Table S3, respectively. After mixing, the mixture was heated to 70°C followed by a linear cooling ramp to 25°C over 50 minutes. The concentration of oligonucleotide staples were 3 times of the m13mp18 scaffold. After annealing, excess oligonucleotides were removed using Amicon Ultra 100kDa filters by washing twice. During each washing step, the above mixture was added to a washing buffer (combination of 460 μL 10mM Tris-HCl (pH=8) and 0.5 mM $MgCl_2$), and centrifuged at 9000 × g for 10 minutes at 4°C. The final sample was then pipetted out and collected from the bottom of the Amicon tube. This usually yielded ~30 μL at a concentration of ~50 ng/uL. All prepared construct samples were stored at 4°C before nanopore measurements.

6. Materials

Commercial reagents used in experiments included nuclease-free water (Ambion, catalog number AM9937), 100 × Tris-EDTA concentrated buffer solution (Sigma-Aldrich, Catalog number T9285), Lithium chloride for molecular biology ≥99% purity (Sigma-Aldrich, Catalog number L9650), Tris-HCl BioPerformance certified, ≥99% purity (Sigma-Aldrich, catalog number T5941). All nanopore-measurement solutions and buffers prepared from these reagents were filtered twice with 0.22 μm Millipore syringe filter units (MF-Merck Millipore™, Catalog number GSWP04700). Streptavidin was purchased from Thermo Scientific™ (Catalog number 21122). Bovine Serum Albumin was purchased from Sangon Biotech (0.5 mg/ml, Catalog number C510069). Immunoglobulin G was purchased from Sangon Biotech (Catalog number D110502). Alpha-fetoprotein Recombinant protein (hFc tag C-Terminus) was purchased from Sangon Biotech (Catalog number D145069).

7. Finite Element Method (FEM) for Analyzing the Two Nanopores

We employed the Finite Element Method (FEM) to analyze the electric field distributions in both nanopore platforms. We constructed models of the glass and SiN_x nanopore systems by solving the coupled Poisson-Nernst-Planck (PNP) and Navier-Stokes (NS) equations. The Poisson equation describes the electric potential,

$$\nabla^2\Phi = -\frac{Z_i e\rho_i}{\epsilon\epsilon_0} = -\left(\frac{Z_i e\rho_0}{\epsilon\epsilon_0}\right)\exp\left(-Z_i e\Phi/kT\right) \quad \text{(S2)}$$

where Φ is the electrical potential, ρ_i is the number concentration of ith ionic species, ρ_0 is the bulk number concentration of that species, and Z_i is the ion valency. The Nernst-Planck equations account for ion transport mechanisms (diffusion, migration, and convection),

$$\rho\left(\frac{\partial \vec{v}}{\partial t} + \vec{v} \cdot \nabla \vec{v}\right) = -\nabla p + \mu \nabla^2 \vec{v} + \vec{F} \tag{S3}$$

where $\vec{v}$ is the velocity of the liquid, p is the hydrostatic pressure, μ is the fluid viscosity, and F is the density of external forces on liquid. The Navier-Stokes equations address fluid dynamics,

$$\frac{\partial \rho_i}{\partial t} = -\nabla \vec{N}_i = -\left(n_i \vec{v} - D_i \nabla \rho_i - \mu_i \rho_i \nabla \emptyset\right) \tag{S4}$$

where $\vec{N}_i$ is the ionic flux density of the ith ionic species, D_i is the diffusivity, and μ_i is the mobility. The coupling of physics was implemented by solving the PNP and NS equations. Specifically, the space charge density calculated from the Nernst-Planck interface was used as the source term in the Poisson equation, and the electric field was used as the volume force driving the Navier-Stokes fluid flow. We used a fully coupled solver that solved all the equations (electric potential, ion concentration, fluid velocity, and pressure) simultaneously in one matrix.

The simulation models for the two nanopores are illustrated in Figure S4. In the simulation, the capillary was simplified as a conical pore with two angles, connected to reservoirs on both sides. As shown in Figure S4a, the parameters H_1, L_1, H_2, and W were set to 10 μm, 10 μm, 1 μm, and 4 μm, respectively. In the magnified view on the right, the parameters L_2, D, θ_1, and θ_2 were 100 nm, 10 nm, 0.092 rad, and 0.046 rad, respectively. For the SiN_x nanopore shown in Figure S4b, the parameters H_3 and H_4 were set to 10.5 μm and 15 nm, respectively.

We performed the FEM simulations using COMSOL Multiphysics. For the inner pore boundaries of both platforms, the mesh element size was refined to 0.1 nm. Consequently, the total number of domain elements is 44,626 for the glass nanopore model and 47,103 for the SiN_x nanopore model. Using the glass nanopore model as an example, we conducted a mesh convergence study by varying the element size at the inner pore surface. The results, shown in Figure S21, demonstrate that the electric field strength profile remains consistent across different mesh densities. This confirms that our selected mesh configuration is optimal.

The boundary conditions of the simulation model are listed in Table S5. The ionic species and concentration in the simulations were set to 4 M LiCl, consistent with experimental conditions. The buffer effects were implicitly modeled by directly assigning surface charge densities to the nanopores: $-35\ mC \cdot m^{-2}$ for glass (SiO_2) and $-20\ mC \cdot m^{-2}$ for SiN_x.

Supplementary Figures

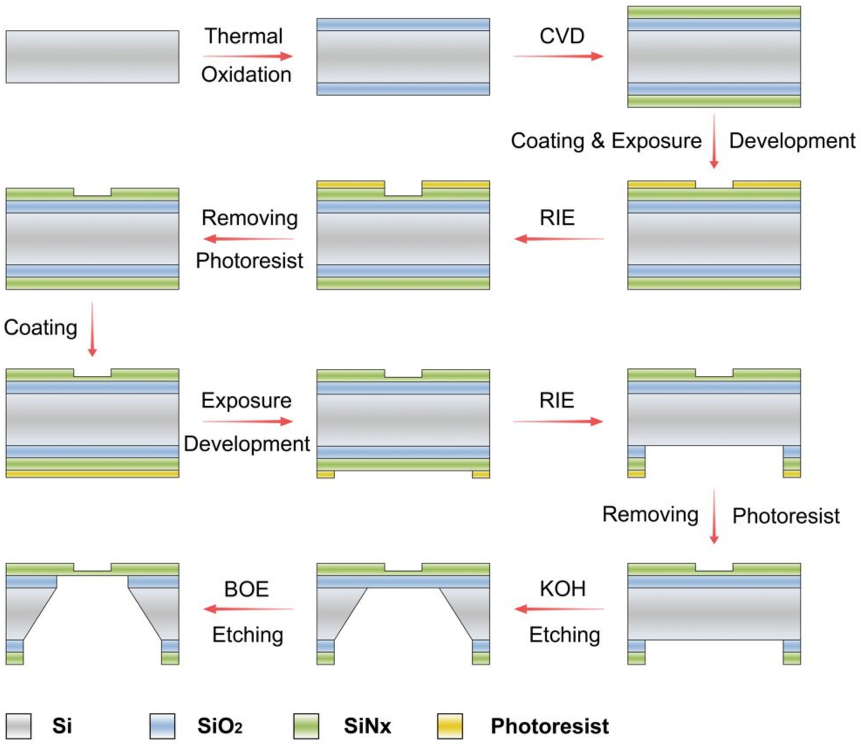

Figure S1. Process flow for wafer preparation. The fabrication steps include thermal oxidation, LPCVD deposition, front-side photoresist coating, exposure, and development, front-side RIE, photoresist removal, back-side photoresist coating, exposure, and development, back-side RIE, photoresist removal, KOH wet etching, and final BOE to release the membrane.

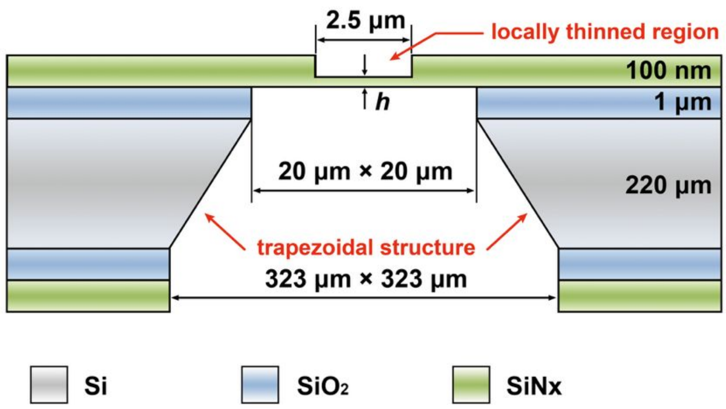

Figure S2. Schematic diagram of the silicon nitride nanopore membrane chip structure, including the silicon nitride layer, silicon oxide layer, and silicon substrate. The diameter of the locally thinned region is $\phi = 2.5$ μm. The thickness of the SiN_x membrane here is h = 15 nm.

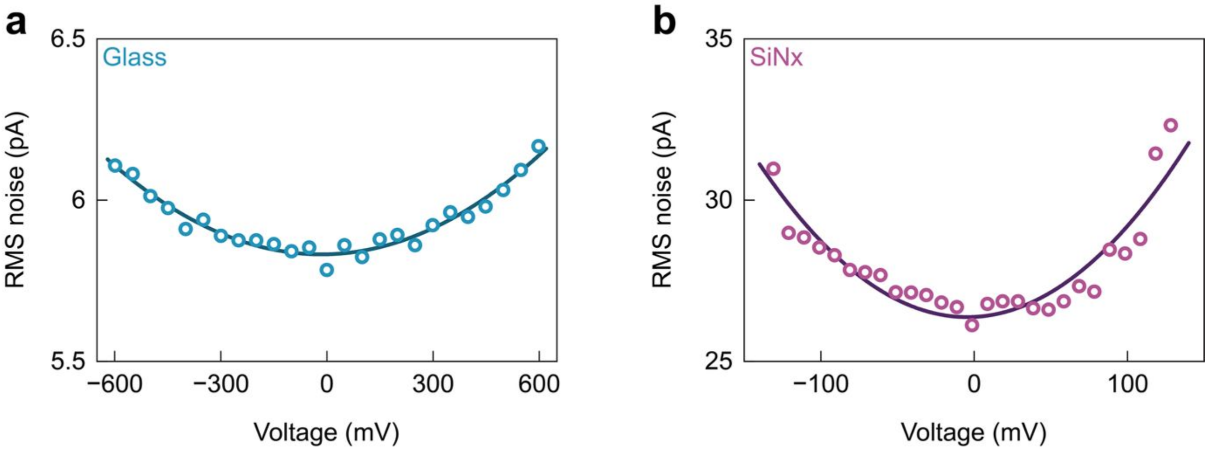

Figure S3. RMS current noise as a function of applied voltage for two nanopore platforms: (a) glass nanopores and (b) SiN_x nanopores. RMS noise values were calculated from 3 s current traces recorded at each applied voltage. The solid lines represent parabolic fits to the data. The current output was low-pass filtered at 50 kHz prior to analysis.

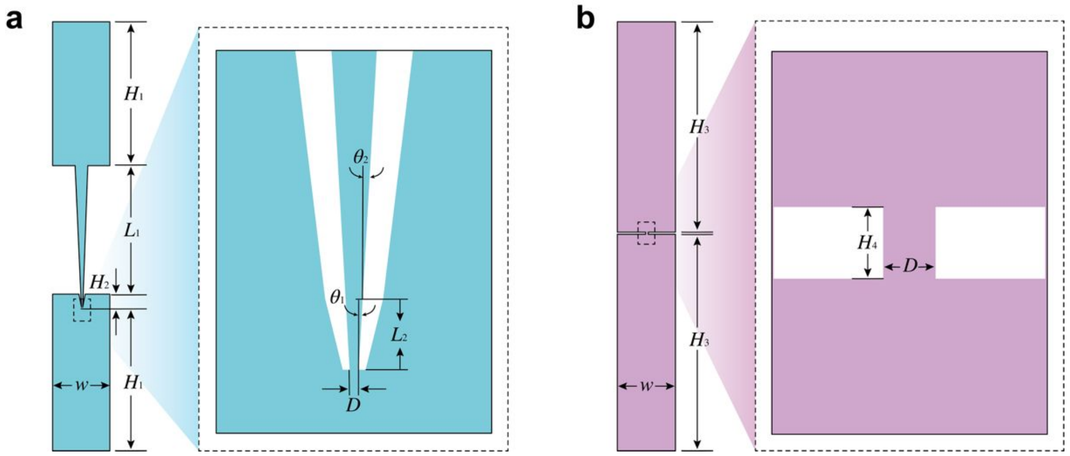

Figure S4. Schematic diagrams of the simulation models used for nanopore calculations. (a) Geometry of the glass nanopore model, simplified as a tapered pore connected to two reservoirs. The parameters are defined as: $H_1 = 10$ µm, $L_1 = 10$ µm, $H_2 = 1$ µm, and W = 4 µm. In the magnified view on the right, the detailed pore dimensions are $L_2 = 100$ nm, D = 10 nm, $\theta_1 = 0.092$ rad, and $\theta_2 = 0.046$ rad. The mesh along the inner pore boundary was refined to 0.1 nm. (b) Geometry of the SiN_x nanopore model, where $H_3 = 10.5$ µm and $H_4 = 15$ nm.

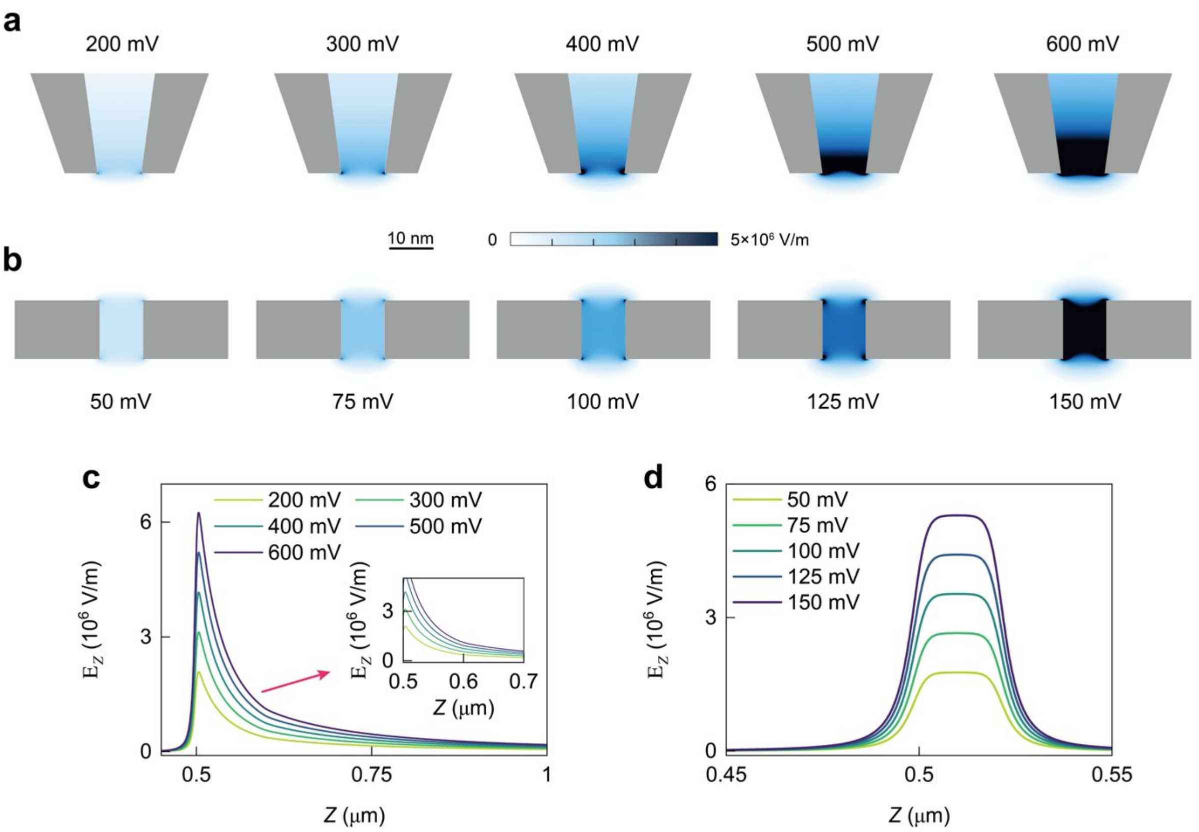

Figure S5. Electric field distribution along the nanopore for two nanopore platforms. Finite element method (FEM) simulation of the electric field strength at (a) applied voltages of 200, 300, 400, 500, and 600 mV for the glass nanopore, and (b) 50, 75, 100, 125, and 150 mV for the SiN_x nanopore, both with a pore diameter of 10 nm. (c) Axial electric field profiles corresponding to the five voltages in the glass nanopore. The inset shows an enlarged view highlighting the extended field decay along the conical nanochannel. (d) Axial electric field profiles for the five voltages in the SiN_x nanopore.

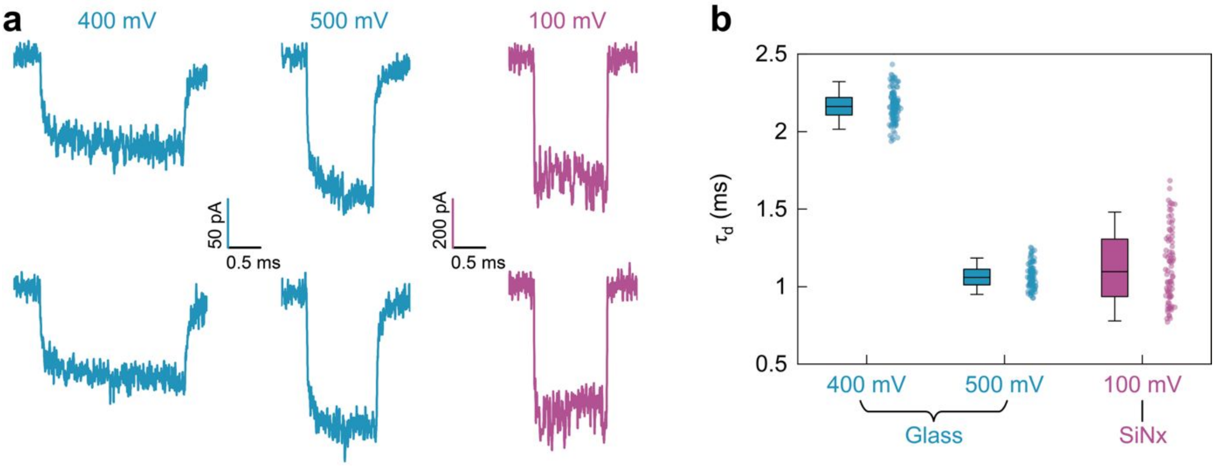

Figure S6. Comparison of DNA translocation times in glass and SiN_x nanopores at different applied voltages. (a) Representative translocation events of dsDNA translocation through a glass nanopore (left, 400 mV and 500 mV) and a SiN_x nanopore (right, 100 mV). (b) Box plot showing the distribution of translocation times (τ_d) for N = 100 events under the three conditions. Median translocation times are 2.16 ms (400 mV, glass), 1.06 ms (500 mV, glass), and 1.10 ms (100 mV, SiN_x). Boxes represent the interquartile range (IQR; 25th-75th percentile), with the central line indicating the median. Whiskers extend to the most extreme points within 1.5 × IQR, and individual points on the right denote single-event data. Blue and magenta indicate glass and SiN_x nanopores, respectively.

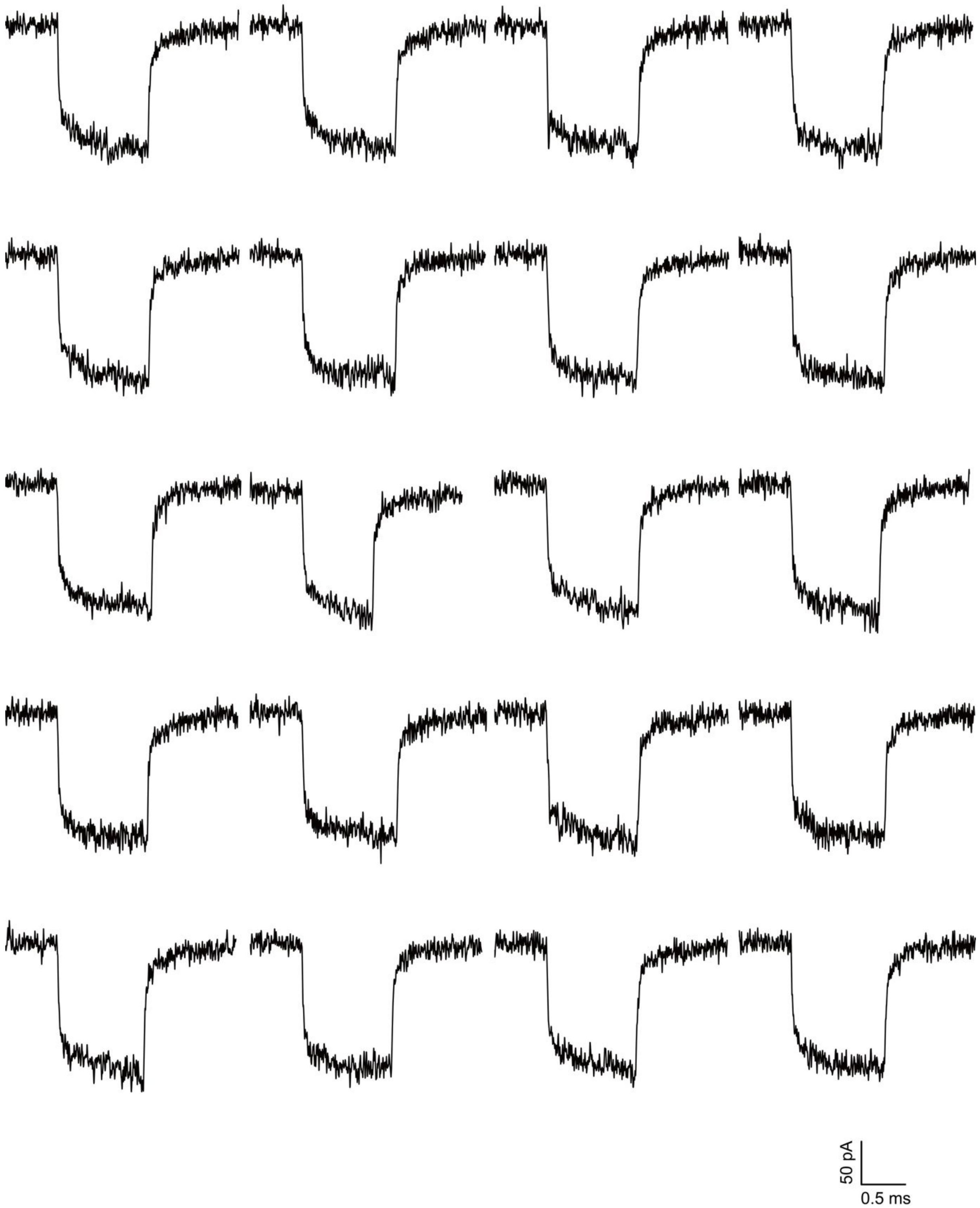

Figure S7. Example translocation events of double-stranded M13mp18 DNA (7.2 kbp) measured in a 10-nm glass nanopore.

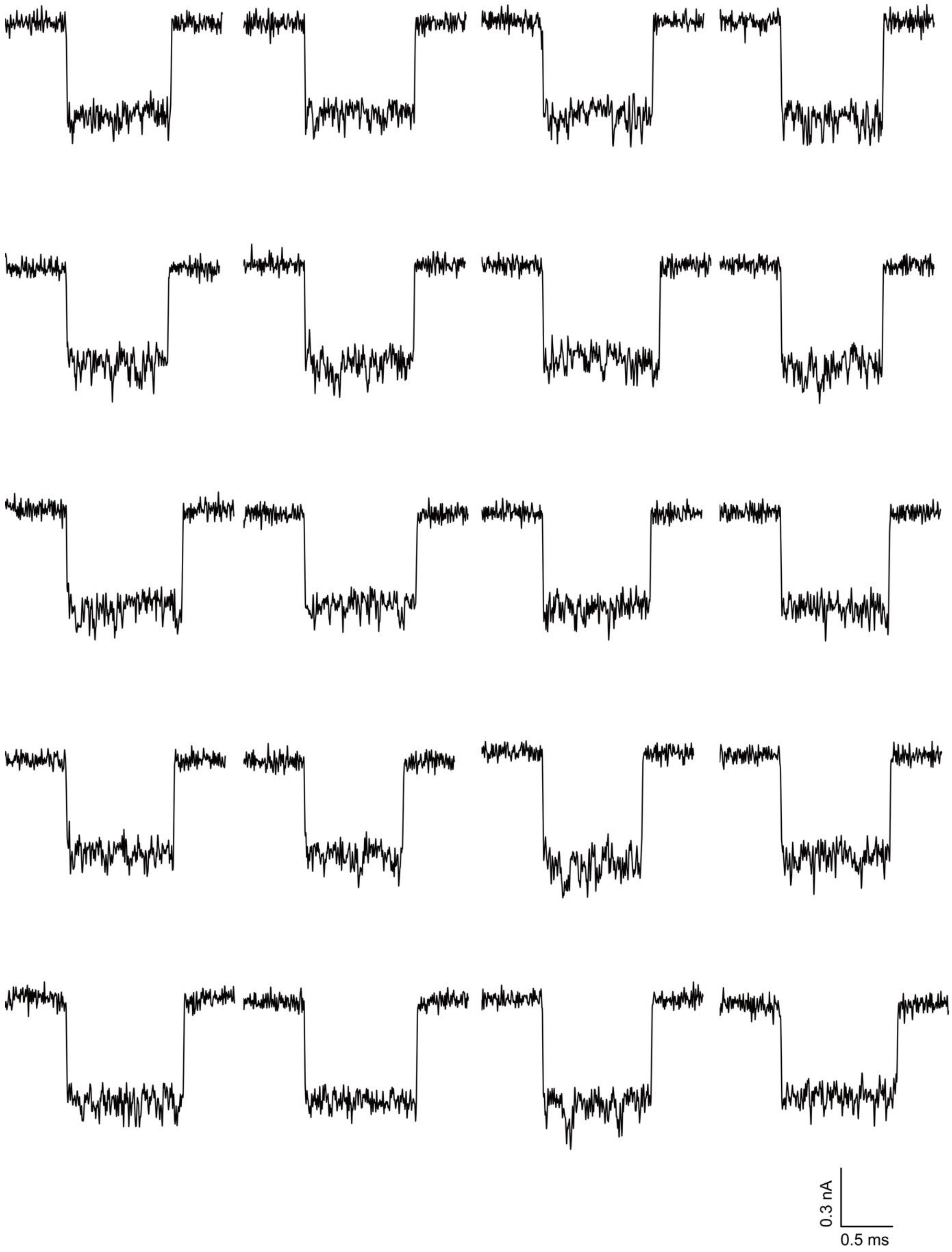

Figure S8. Example translocation events of double-stranded M13mp18 DNA (7.2 kbp) measured in a 10-nm SiN_x nanopore.

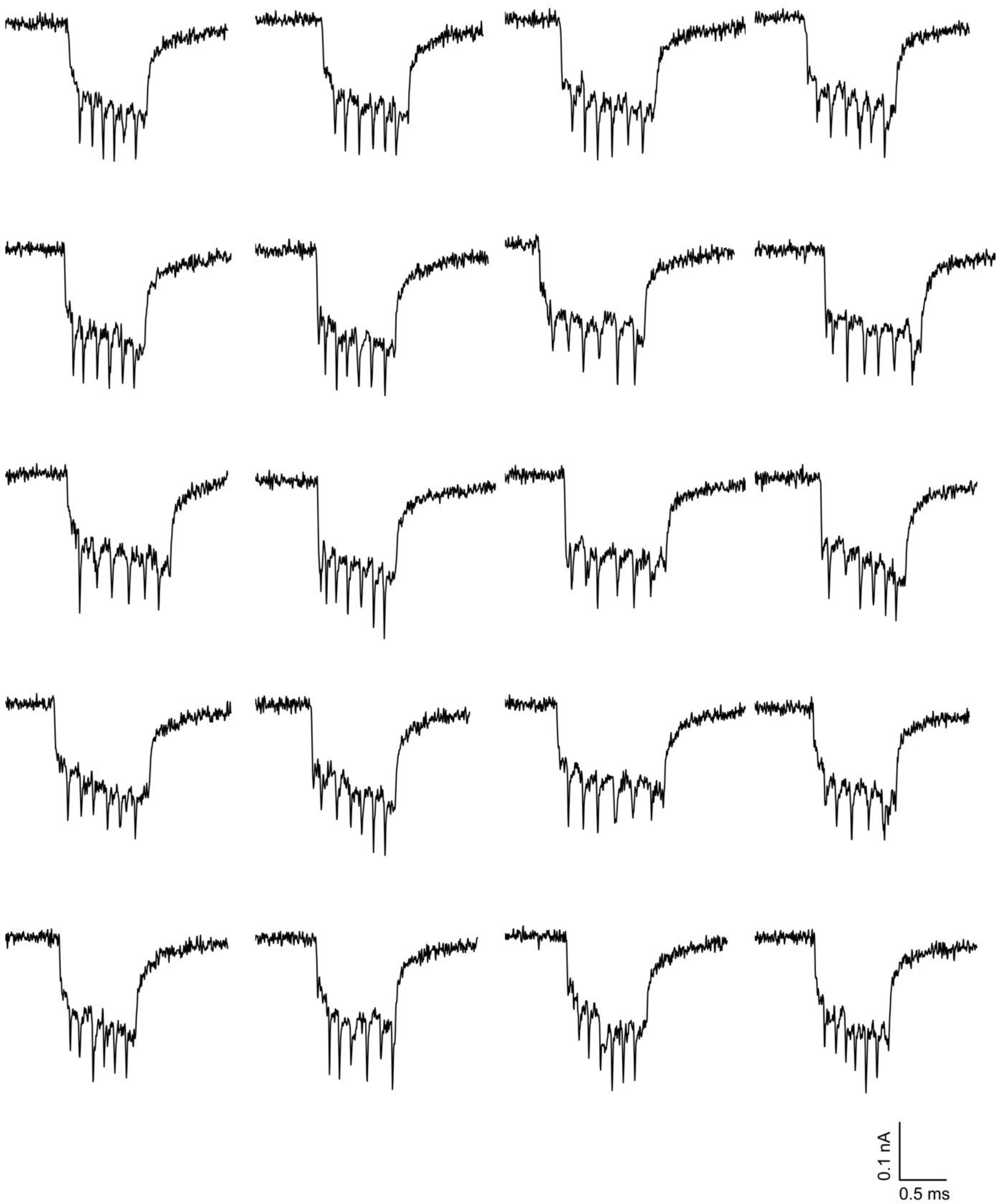

Figure S9. Example translocation events of a DNA construct with six barcoded dumbbell nanostructures measured in a 10-nm glass nanopore.

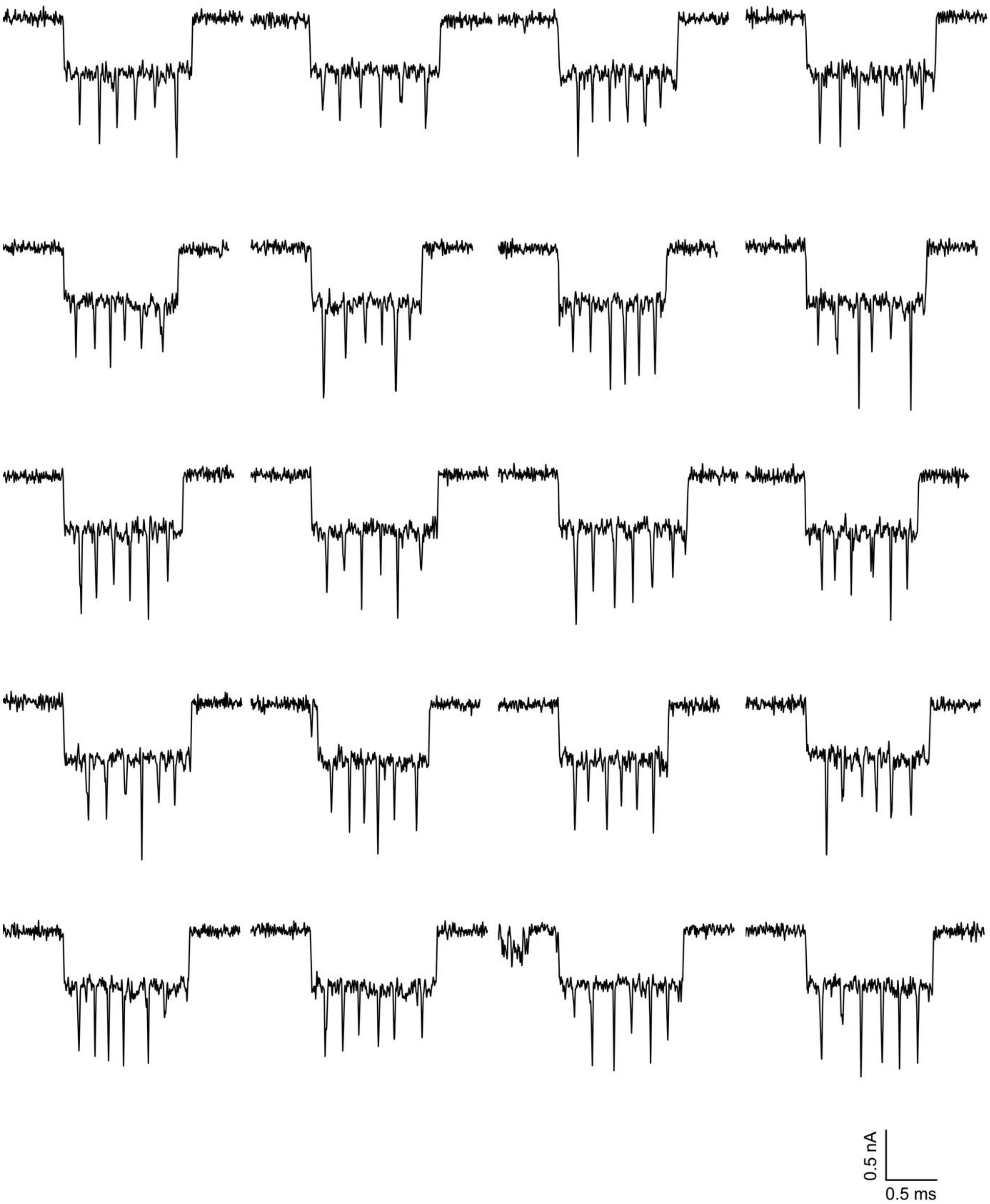

Figure S10. Example translocation events of a DNA construct with six barcoded dumbbell nanostructures measured in a 10-nm SiN_x nanopore.

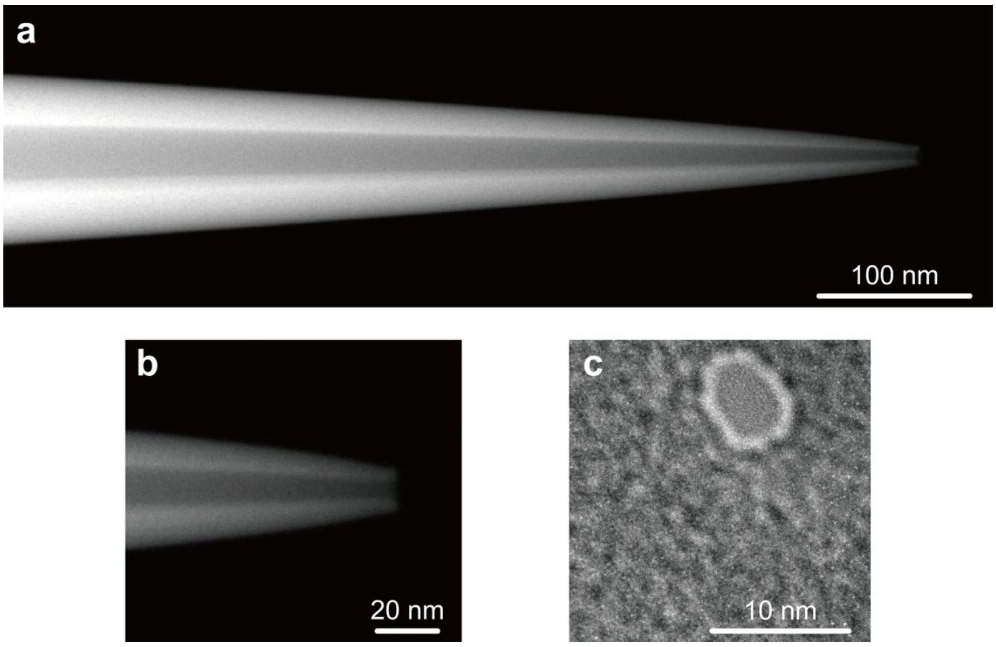

Figure S11. TEM characterization of (a) a 5 nm glass nanopore and (c) a 5 nm SiN_x nanopore. (b) Enlarged view of the glass nanopore shown in (a).

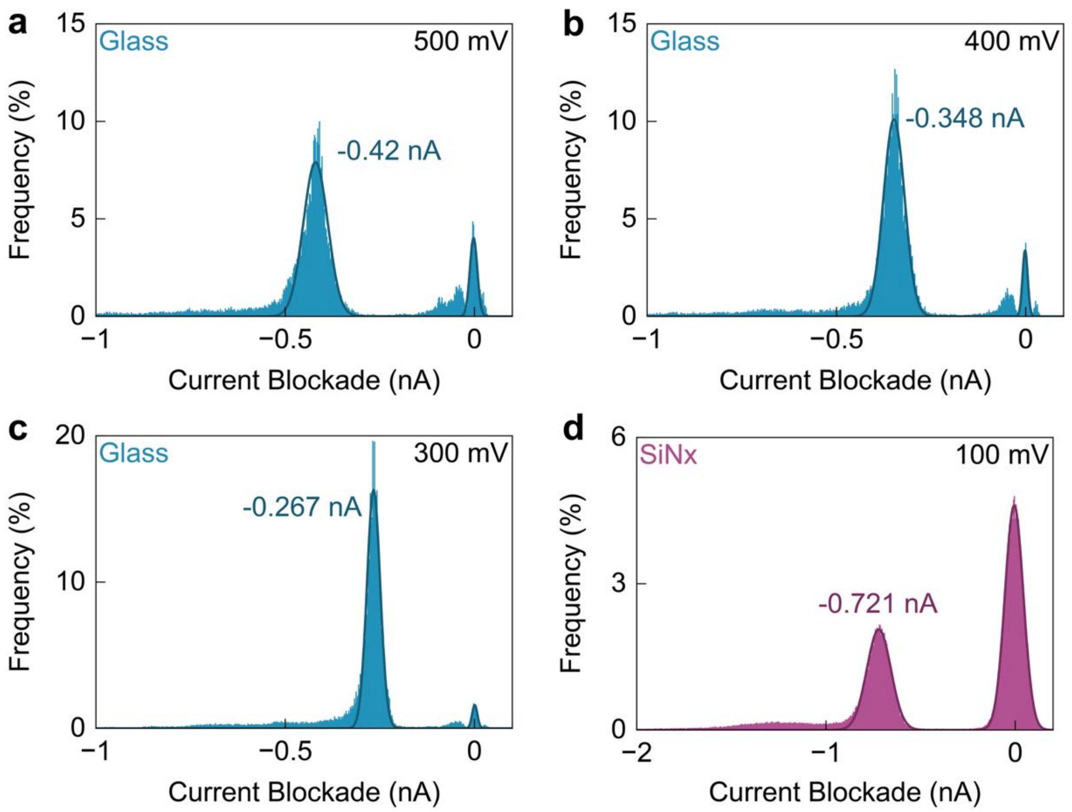

Figure S12. All-points current histograms corresponding to DNA translocation events measured in 5 nm glass and SiN_x nanopores under applied voltages of (a) 500 mV, (b) 400 mV, (c) 300 mV, and (d) 100 mV. The mean current blockade values extracted from Gaussian fits are 0.42, 0.35, 0.27, and 0.72 nA, respectively.

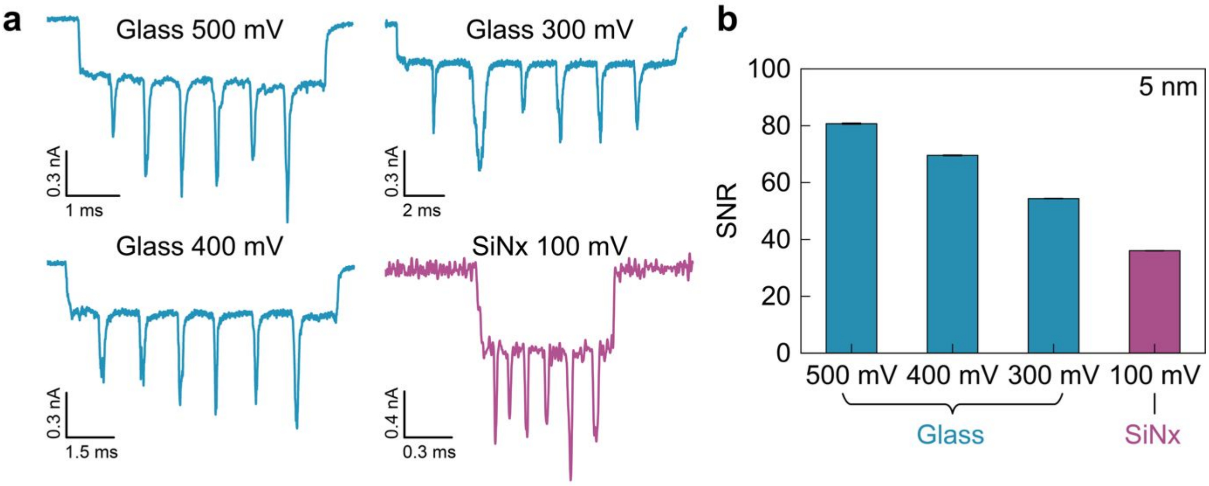

Figure S13. Representative translocation events and corresponding signal-to-noise ratios (SNRs) for 5 nm glass and SiN_x nanopores. (a) Example DNA translocation events recorded in a glass nanopore at applied voltages of 500, 400, and 300 mV and in a SiN_x nanopore at 100 mV. (b) SNR values corresponding to these four conditions. The analyte in all measurements is the DNA construct.

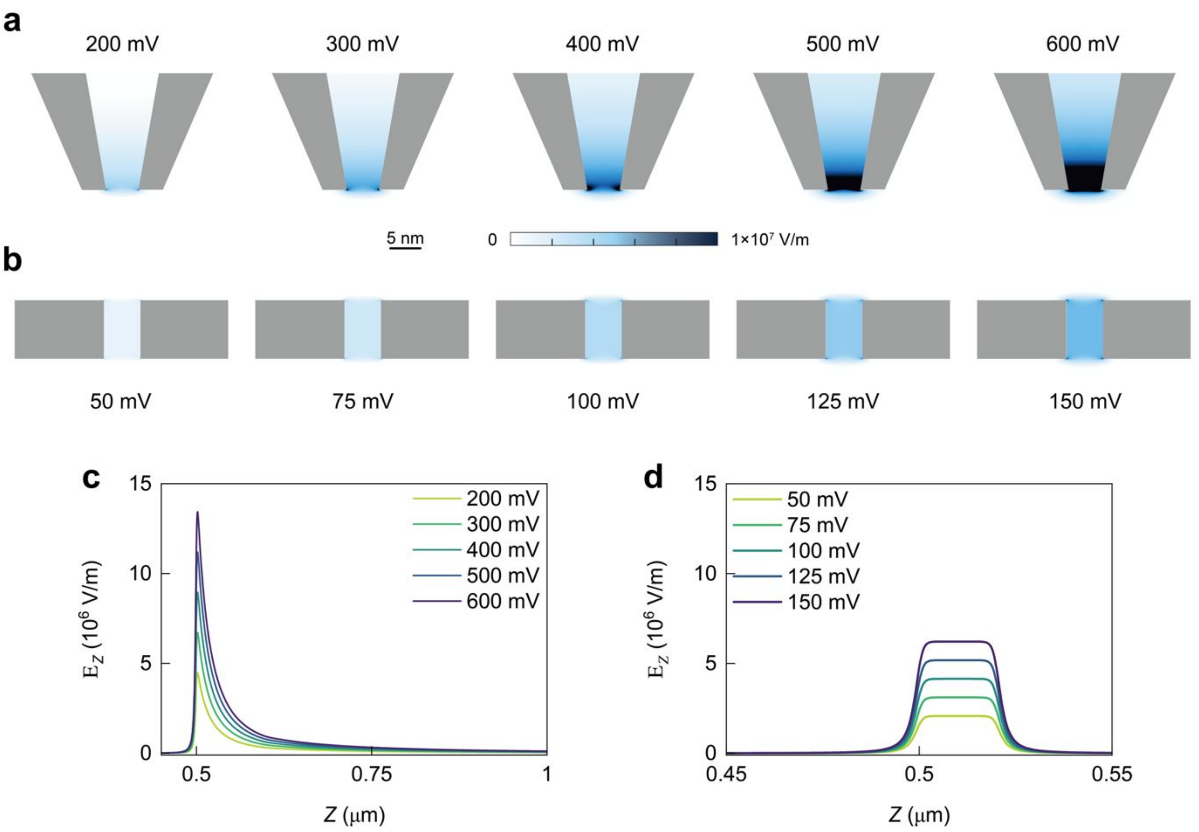

Figure S14. Electric field distribution along the 5-nm nanopore for two nanopore platforms. FEM simulation of the electric field strength at (a) applied voltages of 200, 300, 400, 500, and 600 mV for the glass nanopore, and (b) 50, 75, 100, 125, and 150 mV for the SiN_x nanopore, both with a pore diameter of 5 nm. (c) Axial electric field profiles corresponding to the five voltages in the glass nanopore. (d) Axial electric field profiles for the five voltages in the SiN_x nanopore.

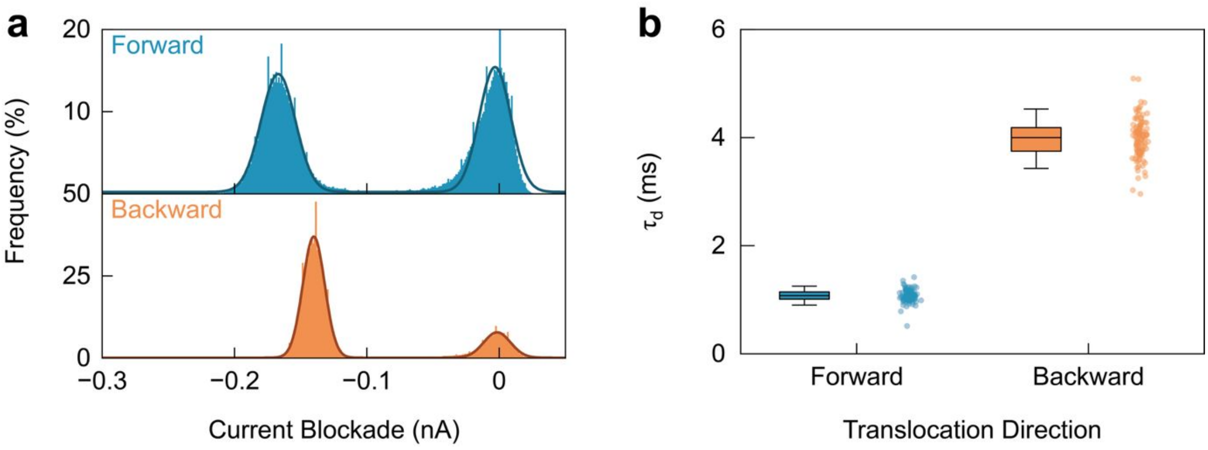

Figure S15. Comparison of forward and backward DNA translocation through a 10 nm glass nanopore. (a) All-points current histograms for forward (top) and backward (bottom) translocation events. The mean current blockade (ΔI) is 0.167 nA for forward and 0.14 nA for backward translocation. (b) Box plot showing the distribution of translocation durations (τ_d) for N = 100 events in each direction. The median τ_d is 1.08 ms for forward translocation and 4 ms for backward translocation. Boxes indicate the interquartile range (25–75%), with the central line denoting the median. Whiskers extend to the most extreme data points within 1.5 × IQR, and individual dots on the right represent single-event data. Blue and orange correspond to forward and backward translocation events, respectively.

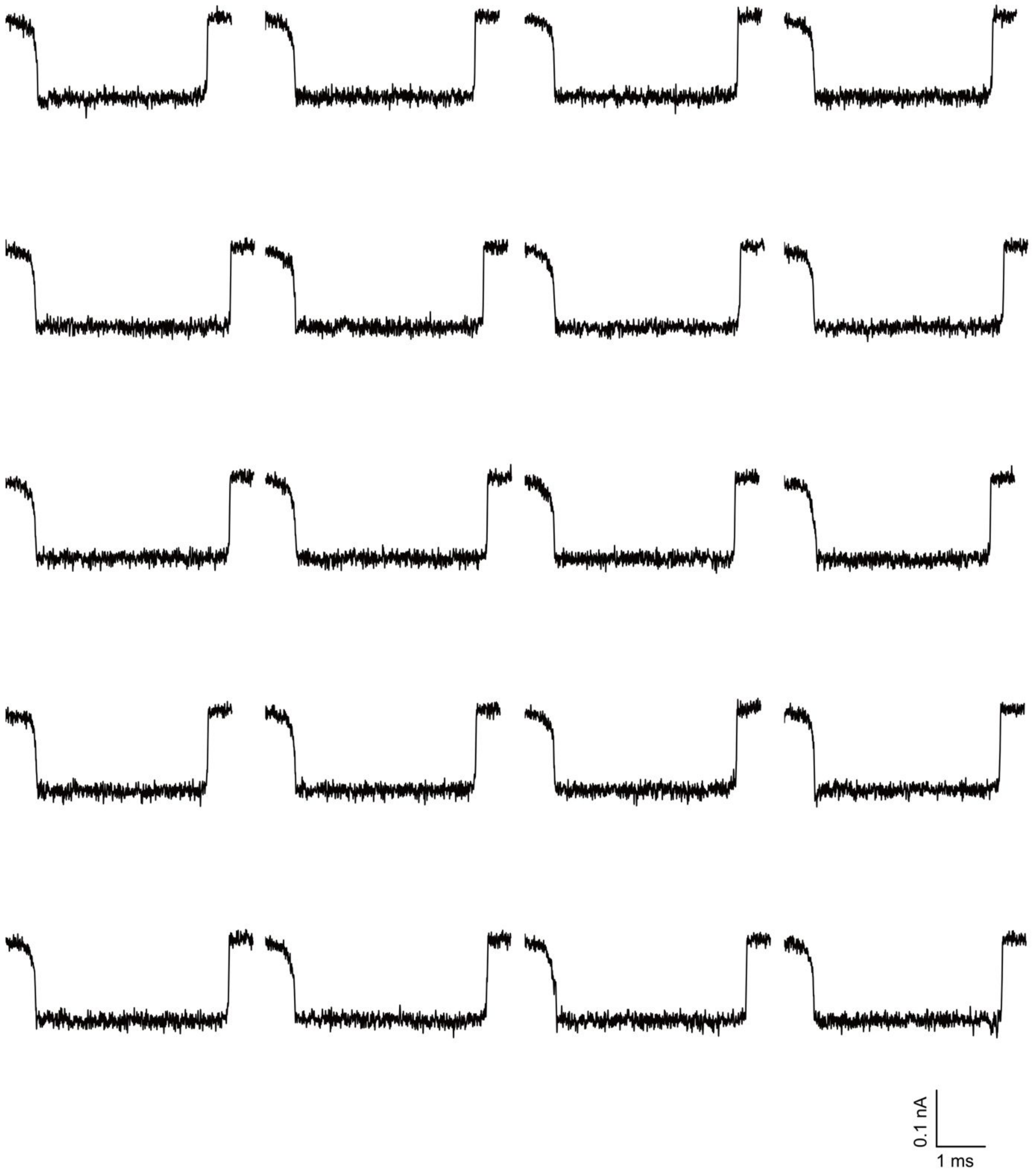

Figure S16. Example backward translocation events of dsDNA molecules measured in a 10-nm glass nanopore.

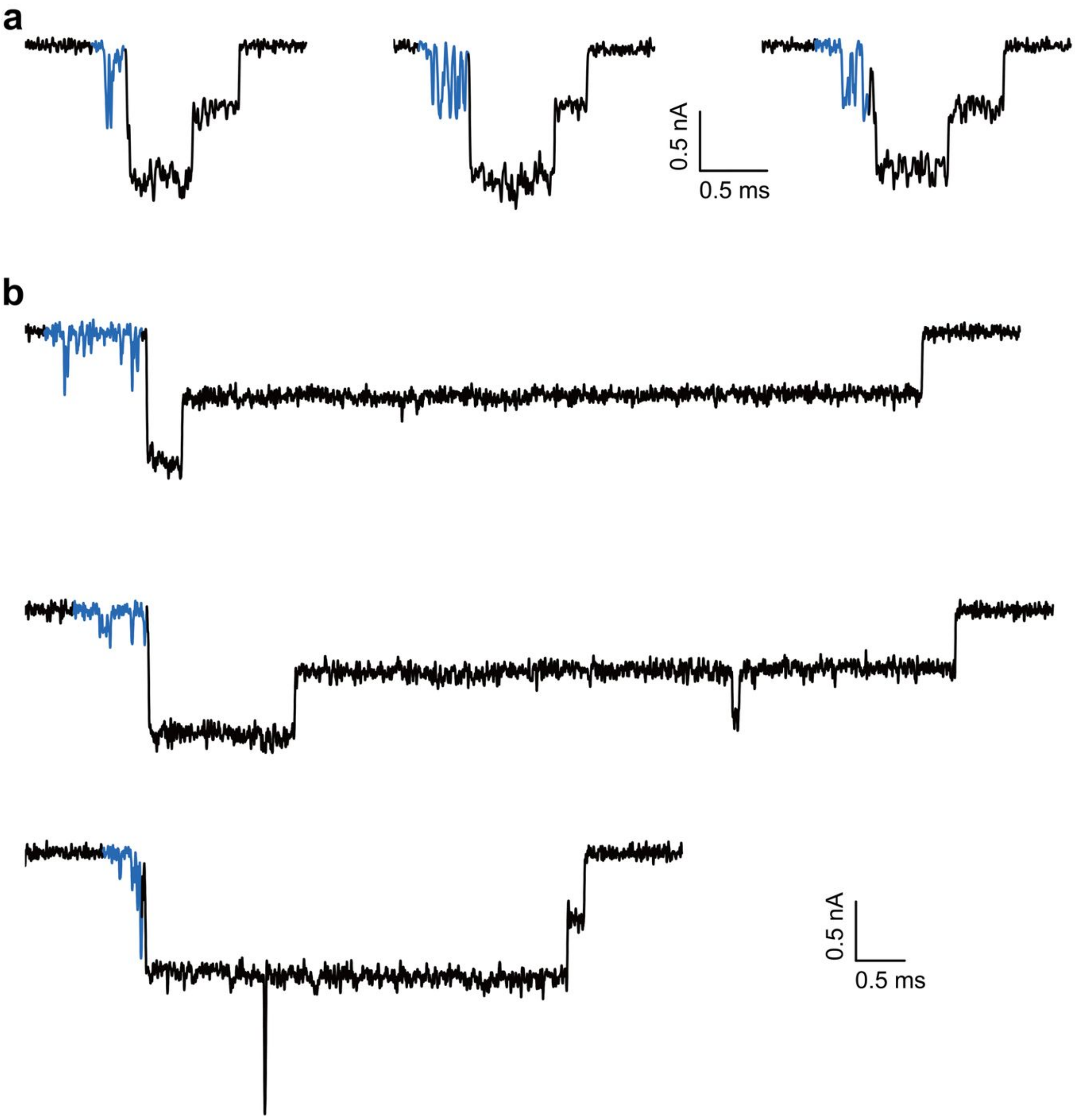

Figure S17. Example perturbed events of (a) dsDNA and (b) λ-DNA measured in a 10-nm SiN_x nanopore.

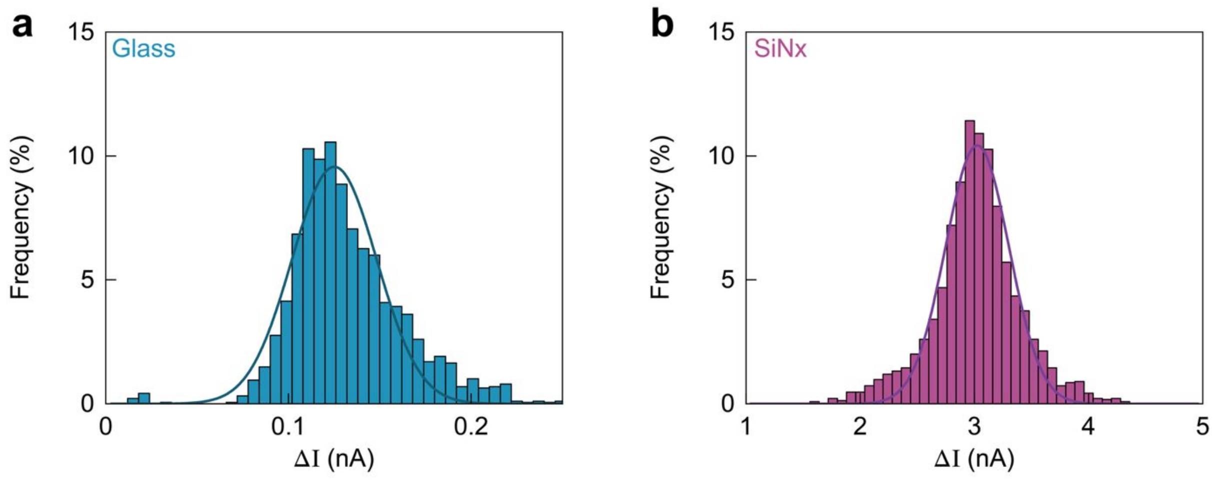

Figure S18. Current-blockade (ΔI) histograms of BSA translocations through (a) a 5-nm glass nanopore and (b) a 5-nm SiN_x nanopore. The mean ΔI values obtained from Gaussian fits are 0.12 nA for the glass nanopore and 3.0 nA for the SiN_x nanopore.

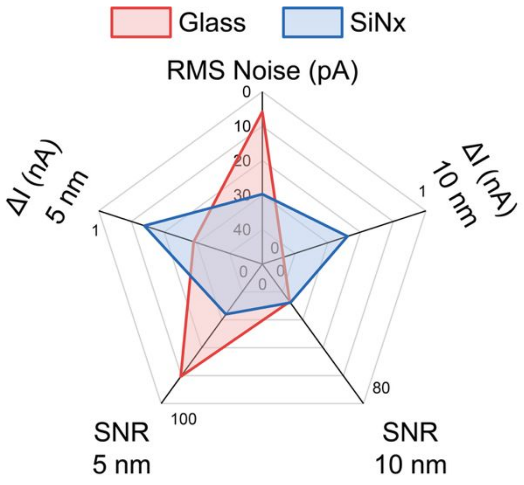

Figure S19. Spider plots summarizing the sensing performance of glass and SiN_x nanopores for DNA molecules measured using 10 nm and 5 nm pores.

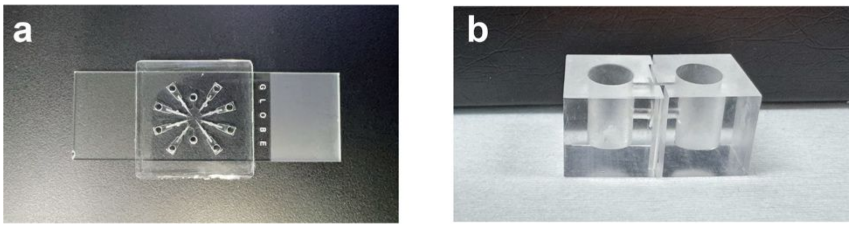

Figure S20. Assemblies of the glass nanopore chip and SiN_x nanopore chip. (a) Glass. (b) SiN_x.

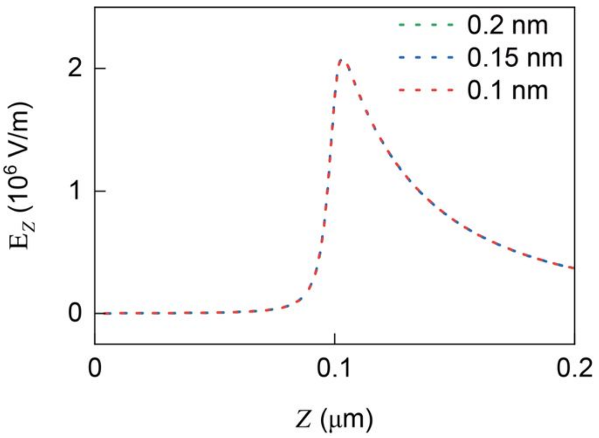

Figure S21. Mesh convergence study of the FEM simulation. The mesh size of the inner pore surface of the glass model is set to 0.1, 0.15, and 0.2 nm, respectively. In the model, the nanopore diameter is set to 10 nm, and the voltage is set to 200 mV.

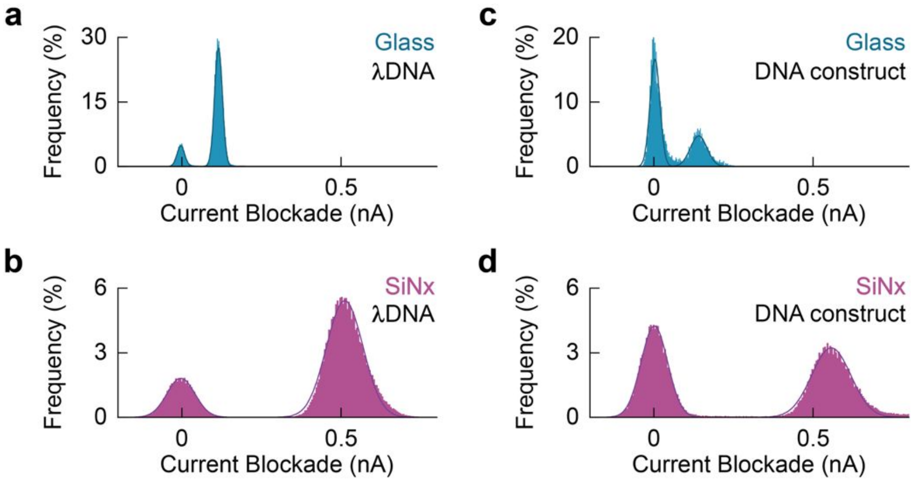

Figure S22. All-points current histograms of linear DNA translocations in 10 nm nanopores, showing baseline and blockade current levels for four cases. (a) λ-DNA measured in glass nanopores. (b) λ-DNA measured in SiN_x nanopores. (c) DNA construct measured in glass nanopores. (d) DNA construct measured in SiN_x nanopores.

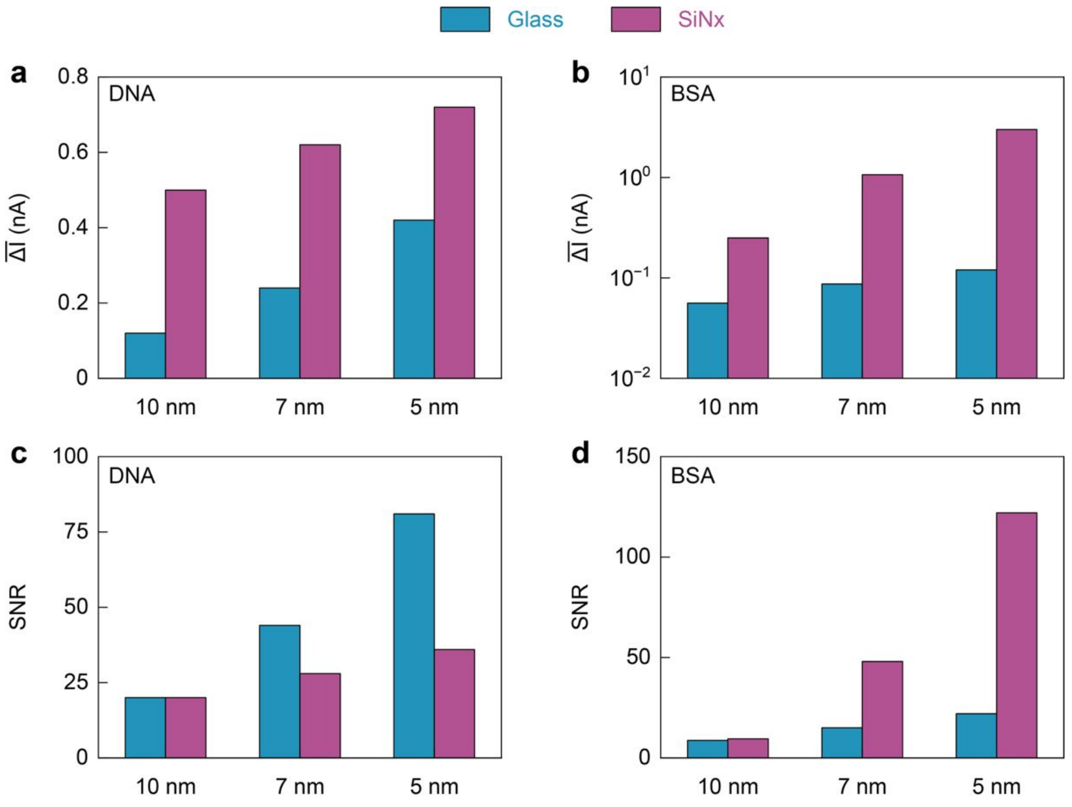

Figure S23. Trends of mean current blockade and SNR with nanopore size in the two nanopore platforms. (a) Mean current blockade of DNA translocation in glass and SiN_x nanopores as a function of nanopore diameter. (b) Mean current blockade of BSA translocation in glass and SiN_x nanopores as a function of nanopore diameter. (c) SNR of DNA translocation in glass and SiN_x nanopores as a function of nanopore diameter. (d) SNR of BSA translocation in glass and SiN_x nanopores as a function of nanopore diameter.

Supplementary Tables

Table S1. Pulling parameters of glass capillaries using P2000/F laser puller.

**Diameter**	**HEAT**	**FIL**	**VEL**	**DEL**	**PUL**
10 ± 2 nm	500	0	25	170	200
5 ± 2 nm	520	0	25	170	200

Table S2. Sequences of the 190 oligonucleotide staples complementary to the single-stranded M13mp18 scaffold.

Oligo No.	Sequence (5' to 3')	Length (nt)
1	TTTTCGTAATCATGGTCATAGCTGTTTCCTGTGTGAAATTGTTATC	46
2	CGCTCACAATTCCACACAACATACGAGCCGGAAGCATA	38
3	AAGTGTAAAGCCTGGGGTGCCTAATGAGTGAGCTAACT	38
4	CACATTAATTGCGTTGCGCTCACTGCCCGCTTTCCAGT	38
5	CGGGAAACCTGTCGTGCCAGCTGCATTAATGAATCGGC	38
6	CAACGCGCGGGGAGAGGCGGTTTGCGTATTGGGCGCCA	38
7	GGGTGGTTTTTCTTTTCACCAGTGAGACGGGCAACAGC	38
8	TGATTGCCCTTCACCGCCTGGCCCTGAGAGAGTTGCAG	38
9	CAAGCGGTCCACGCTGGTTTGCCCCAGCAGGCGAAAAT	38
10	CCTGTTTGATGGTGGTTCCGAAATCGGCAAAATCCCTT	38
11	ATAAATCAAAAGAATAGCCCGAGATAGGGTTGAGTGTT	38
12	GTTCCAGTTTGGAACAAGAGTCCACTATTAAAGAACGT	38
13	GGACTCCAACGTCAAAGGGCGAAAAACCGTCTATCAGG	38
14	GCGATGGCCCACTACGTGAACCATCACCCAAATCAAGT	38
15	TTTTTGGGGTCGAGGTGCCGTAAAGCACTAAATCGGAA	38
16	CCCTAAAGGGAGCCCCCGATTTAGAGCTTGACGGGGAA	38
17	AGCCGGCGAACGTGGCGAGAAAGGAAGGGAAGAAAGCG	38
18	AAAGGAGCGGGCGCTAGGGCGCTGGCAAGTGTAGCGGT	38
19	CACGCTGCGCGTAACCACCACACCCGCCGCGCTTAATG	38
20	CGCCGCTACAGGGCGCGTACTATGGTTGCTTTGACGAG	38
21	CACGTATAACGTGCTTTCCTCGTTAGAATCAGAGCGGG	38
22	AGCTAAACAGGAGGCCGATTAAAGGGATTTTAGACAGG	38
23	AACGGTACGCCAGAATCCTGAGAAGTGTTTTTATAATC	38
24	AGTGAGGCCACCGAGTAAAAGAGTCTGTCCATCACGCA	38
25	AATTAACCGTTGTAGCAATACTTCTTTGATTAGTAATA	38
26	ACATCACTTGCCTGAGTAGAAGAACTCAAACTATCGGC	38
27	CTTGCTGGTAATATCCAGAACAATATTACCGCCAGCCA	38
28	TTGCAACAGGAAAAACGCTCATGGAAATACCTACATTT	38
29	TGACGCTCAATCGTCTGAAATGGATTATTTACATTGGC	38
30	AGATTCACCAGTCACACGACCAGTAATAAAAGGGACAT	38
31	TCTGGCCAACAGAGATAGAACCCTTCTGACCTGAAAGC	38
32	GTAAGAATACGTGGCACAGACAATATTTTTGAATGGCT	38
33	ATTAGTCTTTAATGCGCGAACTGATAGCCCTAAAACAT	38
34	CGCCATTAAAAATACCGAACGAACCACCAGCAGAAGAT	38
35	AAAACAGAGGTGAGGCGGTCAGTATTAACACCGCCTGC	38
36	AACAGTGCCACGCTGAGAGCCAGCAGCAAATGAAAAAT	38
37	CTAAAGCATCACCTTGCTGAACCTCAAATATCAAACCC	38
38	TCAATCAATATCTGGTCAGTTGGCAAATCAACAGTTGA	38
39	AAGGAATTGAGGAAGGTTATCTAAAATATCTTTAGGAG	38
40	CACTAACAACTAATAGATTAGAGCCGTCAATAGATAAT	38
41	ACATTTGAGGATTTAGAAGTATTAGACTTTACAAACAA	38
42	TTCGACAACTCGTATTAAATCCTTTGCCCGAACGTTAT	38
43	TAATTTTAAAAGTTTGAGTAACATTATCATTTTGCGGA	38
44	ACAAAGAAACCACCAGAAGGAGCGGAATTATCATCATA	38
45	TTCCTGATTATCAGATGATGGCAATTCATCAATATAAT	38
46	CCTGATTGTTTGGATTATACTTCTGAATAATGGAAGGG	38
47	TTAGAACCTACCATATCAAAATTATTTGCACGTAAAAC	38
48	AGAAATAAAGAAATTGCGTAGATTTTCAGGTTTAACGT	38
49	CAGATGAATATACAGTAACAGTACCTTTTACATCGGGA	38
50	GAAACAATAACGGATTCGCCTGATTGCTTTGAATACCA	38
51	AGTTACAAAATCGCGCAGAGGCGAATTATTCATTTCAA	38
52	TTACCTGAGCAAAAGAAGATGATGAAACAAACATCAAG	38
53	AAAACAAAATTAATTACATTTAACAATTTCATTTGAAT	38
54	TACCTTTTTTAATGGAAACAGTACATAAATCAATATAT	38
55	GTGAGTGAATAACCTTGCTTCTGTAAATCGTCGCTATT	38
56	AATTAATTTTCCCTTAGAATCCTTGAAAACATAGCGAT	38
57	AGCTTAGATTAAGACGCTGAGAAGAGTCAATAGTGAAT	38
58	TTATCAAAATCATAGGTCTGAGAGACTACCTTTTTAAC	38

59	CTCCGGCTTAGGTTGGGTTATATAACTATATGTAAATG	38
60	CTGATGCAAATCCAATCGCAAGACAAAGAACGCGAGAA	38
61	AACTTTTTCAAATATATTTTAGTTAATTTCATCTTCTG	38
62	ACCTAAATTTAATGGTTTGAAATACCGACCGTGTGATA	38
63	AATAAGGCGTTAAATAAGAATAAACACCGGAATCATAA	38
64	TTACTAGAAAAAGCCTGTTTAGTATCATATGCGTTATA	38
65	CAAATTCTTACCAGTATAAAGCCAACGCTCAACAGTAG	38
66	GGCTTAATTGAGAATCGCCATATTTAACAACGCCAACA	38
67	TGTAATTTAGGCAGAGGCATTTTCGAGCCAGTAATAAG	38
68	AGAATATAAAGTACCGACAAAAGGTAAAGTAATTCTGT	38
69	CCAGACGACGACAATAAACAACATGTTCAGCTAATGCA	38
70	GAACGCGCCTGTTTATCAACAATAGATAAGTCCTGAAC	38
71	AAGAAAAATAATATCCCATCCTAATTTACGAGCATGTA	38
72	GAAACCAATCAATAATCGGCTGTCTTTCCTTATCATTC	38
73	CAAGAACGGGTATTAAACCAAGTACCGCACTCATCGAG	38
74	AACAAGCAAGCCGTTTTTATTTTCATCGTAGGAATCAT	38
75	TACCGCGCCCAATAGCAAGCAAATCAGATATAGAAGGC	38
76	TTATCCGGTATTCTAAGAACGCGAGGCGTTTTAGCGAA	38
77	CCTCCCGACTTGCGGGAGGTTTTGAAGCCTTAAATCAA	38
78	GATTAGTTGCTATTTTGCACCCAGCTACAATTTTATCC	38
79	TGAATCTTACCAACGCTAACGAGCGTCTTTCCAGAGCC	38
80	TAATTTGCCAGTTACAAAATAAACAGCCATATTATTTA	38
81	TCCCAATCCAAATAAGAAACGATTTTTTGTTTAACGTC	38
82	AAAAATGAAAATAGCAGCCTTTACAGAGAGAATAACAT	38
83	AAAAACAGGGAAGCGCATTAGACGGGAGAATTAACTGA	38
84	ACACCCTGAACAAAGTCAGAGGGTAATTGAGCGCTAAT	38
85	ATCAGAGAGATAACCCACAAGAATTGAGTTAAGCCCAA	38
86	TAATAAGAGCAAGAAACAATGAAATAGCAATAGCTATC	38
87	TTACCGAAGCCCTTTTTAAGAAAAGTAAGCAGATAGCC	38
88	GAACAAAGTTACCAGAAGGAAACCGAGGAAACGCAATA	38
89	ATAACGGAATACCCAAAAGAACTGGCATGATTAAGACT	38
90	CCTTATTACGCAGTATGTTAGCAAACGTAGAAAATACA	38
91	TACATAAAGGTGGCAACATATAAAAGAAACGCAAAGAC	38
92	ACCACGGAATAAGTTTATTTTGTCACAATCAATAGAAA	38
93	ATTCATATGGTTTACCAGCGCCAAAGACAAAAGGGCGA	38
94	CATTCAACCGATTGAGGGAGGGAAGGTAAATATTGACG	38
95	GAAATTATTCATTAAAGGTGAATTATCACCGTCACCGA	38
96	CTTGAGCCATTTGGGAATTAGAGCCAGCAAAATCACCA	38
97	GTAGCACCATTACCATTAGCAAGGCCGGAAACGTCACC	38
98	AATGAAACCATCGATAGCAGCACCGTAATCAGTAGCGA	38
99	CAGAATCAAGTTTGCCTTTAGCGTCAGACTGTAGCGCG	38
100	TTTTCATCGGCATTTTCGGTCATAGCCCCCTTATTAGC	38
101	GTTTGCCATCTTTTCATAATCAAAATCACCGGAACCAG	38
102	AGCCACCACCGGAACCGCCTCCCTCAGAGCCGCCACCC	38
103	TCAGAACCGCCACCCTCAGAGCCACCACCCTCAGAGCC	38
104	GCCACCAGAACCACCACCAGAGCCGCCGCCAGCATTGA	38
105	CAGGAGGTTGAGGCAGGTCAGACGATTGGCCTTGATAT	38
106	TCACAAACAAATAAATCCTCATTAAAGCCAGAATGGAA	38
107	AGCGCAGTCTCTGAATTTACCGTTCCAGTAAGCGTCAT	38
108	ACATGGCTTTTGATGATACAGGAGTGTACTGGTAATAA	38
109	GTTTTAACGGGGTCAGTGCCTTGAGTAACAGTGCCCGT	38
110	ATAAACAGTTAATGCCCCCTGCCTATTTCGGAACCTAT	38
111	TATTCTGAAACATGAAAGTATTAAGAGGCTGAGACTCC	38
112	TCAAGAGAAGGATTAGGATTAGCGGGGTTTTGCTCAGT	38
113	ACCAGGCGGATAAGTGCCGTCGAGAGGGTTGATATAAG	38
114	TATAGCCCGGAATAGGTGTATCACCGTACTCAGGAGGT	38
115	TTAGTACCGCCACCCTCAGAACCGCCACCCTCAGAACC	38
116	GCCACCCTCAGAGCCACCACCCTCATTTTCAGGGATAG	38
117	CAAGCCCAATAGGAACCCATGTACCGTAACACTGAGTT	38
118	TCGTCACCAGTACAAACTACAACGCCTGTAGCATTCCA	38
119	CAGACAGCCCTCATAGTTAGCGTAACGATCTAAAGTTT	38
120	TGTCGTCTTTCCAGACGTTAGTAAATGAATTTTCTGTA	38
121	TGGGATTTTGCTAAACAACTTTCAACAGTTTCAGCGGA	38

122	GTGAGAATAGAAAGGAACAACTAAAGGAATTGCGAATA	38
123	ATAATTTTTTCACGTTGAAAATCTCCAAAAAAAAGGCT	38
124	CCAAAAGGAGCCTTTAATTGTATCGGTTTATCAGCTTG	38
125	CTTTCGAGGTGAATTTCTTAAACAGCTTGATACCGATA	38
126	GTTGCGCCGACAATGACAACAACCATCGCCCACGCATA	38
127	ACCGATATATTCGGTCGCTGAGGCTTGCAGGGAGTTAA	38
128	AGGCCGCTTTTGCGGGATCGTCACCCTCAGCAGCGAAA	38
129	GACAGCATCGGAACGAGGGTAGCAACGGCTACAGAGGC	38
130	TTTGAGGACTAAAGACTTTTTCATGAGGAAGTTTCCAT	38
131	TAAACGGGTAAAATACGTAATGCCACTACGAAGGCACC	38
132	AACCTAAAACGAAAGAGGCAAAAGAATACACTAAAACA	38
133	CTCATCTTTGACCCCCAGCGATTATACCAAGCGCGAAA	38
134	CAAAGTACAACGGAGATTTGTATCATCGCCTGATAAAT	38
135	TGTGTCGAAATCCGCGACCTGCTCCATGTTACTTAGCC	38
136	GGAACGAGGCGCAGACGGTCAATCATAAGGGAACCGAA	38
137	CTGACCAACTTTGAAAGAGGACAGATGAACGGTGTACA	38
138	GACCAGGCGCATAGGCTGGCTGACCTTCATCAAGAGTA	38
139	ATCTTGACAAGAACCGGATATTCATTACCCAAATCAAC	38
140	GTAACAAAGCTGCTCATTCAGTGAATAAGGCTTGCCCT	38
141	GACGAGAAACACCAGAACGAGTAGTAAATTGGGCTTGA	38
142	GATGGTTTAATTTCAACTTTAATCATTGTGAATTACCT	38
143	TATGCGATTTTAAGAACTGGCTCATTATACCAGTCAGG	38
144	ACGTTGGGAAGAAAAATCTACGTTAATAAAACGAACTA	38
145	ACGGAACAACATTATTACAGGTAGAAAGATTCATCAGT	38
146	TGAGATTTAGGAATACCACATTCAACTAATGCAGATAC	38
147	ATAACGCCAAAAGGAATTACGAGGCATAGTAAGAGCAA	38
148	CACTATCATAACCCTCGTTTACCAGACGACGATAAAAA	38
149	CCAAAATAGCGAGAGGCTTTTGCAAAAGAAGTTTTGCC	38
150	AGAGGGGGTAATAGTAAAATGTTTAGACTGGATAGCGT	38
151	CCAATACTGCGGAATCGTCATAAATATTCATTGAATCC	38
152	CCCTCAAATGCTTTAAACAGTTCAGAAAACGAGAATGA	38
153	CCATAAATCAAAAATCAGGTCTTTACCCTGACTATTAT	38
154	AGTCAGAAGCAAAGCGGATTGCATCAAAAAGATTAAGA	38
155	GGAAGCCCGAAAGACTTCAAATATCGCGTTTTAATTCG	38
156	AGCTTCAAAGCGAACCAGACCGGAAGCAAACTCCAACA	38
157	GGTCAGGATTAGAGAGTACCTTTAATTGCTCCTTTTGA	38
158	TAAGAGGTCATTTTTGCGGATGGCTTAGAGCTTAATTG	38
159	CTGAATATAATGCTGTAGCTCAACATGTTTTAAATATG	38
160	CAACTAAAGTACGGTGTCTGGAAGTTTCATTCCATATA	38
161	ACAGTTGATTCCCAATTCTGCGAACGAGTAGATTTAGT	38
162	TTGACCATTAGATACATTTCGCAAATGGTCAATAACCT	38
163	GTTTAGCTATATTTTCATTTGGGGCGCGAGCTGAAAAG	38
164	GTGGCATCAATTCTACTAATAGTAGTAGCATTAACATC	38
165	CAATAAATCATACAGGCAAGGCAAAGAATTAGCAAAAT	38
166	TAAGCAATAAAGCCTCAGAGCATAAAGCTAAATCGGTT	38
167	GTACCAAAAACATTATGACCCTGTAATACTTTTGCGGG	38
168	AGAAGCCTTTATTTCAACGCAAGGATAAAAATTTTTAG	38
169	AACCCTCATATATTTTAAATGCAATGCCTGAGTAATGT	38
170	GTAGGTAAAGATTCAAAAGGGTGAGAAAGGCCGGAGAC	38
171	AGTCAAATCACCATCAATATGATATTCAACCGTTCTAG	38
172	CTGATAAATTAATGCCGGAGAGGGTAGCTATTTTTGAG	38
173	AGATCTACAAAGGCTATCAGGTCATTGCCTGAGAGTCT	38
174	GGAGCAAACAAGAGAATCGATGAACGGTAATCGTAAAA	38
175	CTAGCATGTCAATCATATGTACCCCGGTTGATAATCAG	38
176	AAAAGCCCCAAAAACAGGAAGATTGTATAAGCAAATAT	38
177	TTAAATTGTAAACGTTAATATTTTGTTAAAATTCGCAT	38
178	TAAATTTTTGTTAAATCAGCTCATTTTTTAACCAATAG	38
179	GAACGCCATCAAAAATAATTCGCGTCTGGCCTTCCTGT	38
180	AGCCAGCTTTCATCAACATTAAATGTGAGCGAGTAACA	38
181	ACCCGTCGGATTCTCCGTGGGAACAAACGGCGGATTGA	38
182	CCGTAATGGGATAGGTCACGTTGGTGTAGATGGGCGCA	38
183	TCGTAACCGTGCATCTGCCAGTTTGAGGGGACGACGAC	38
184	AGTATCGGCCTCAGGAAGATCGCACTCCAGCCAGCTTT	38

185	CCGGCACCGCTTCTGGTGCCGGAAACCAGGCAAAGCGC	38
186	CATTCGCCATTCAGGCTGCGCAACTGTTGGGAAGGGCG	38
187	ATCGGTGCGGGCCTCTTCGCTATTACGCCAGCTGGCGA	38
188	AAGGGGGATGTGCTGCAAGGCGATTAAGTTGGGTAACG	38
189	CCAGGGTTTTCCCAGTCACGACGTTGTAAAACGACGGC	38
190	CAGTGCCAAGCTTGCATGCCTGCAGGTCGACTCTAGAGGATCTTTT	46

Table S3. Sequences of the oligonucleotides (six markers) to attach 6×8 dumbbells to the DNA construct.

Group of dumbbells	Sequence (5’ to 3’)	Oligo to replace
A	ACATCACTTGTCCTCTTTTGAGGAACAAGTTTTCTTGTCCTGAGTAGA	26-30
	AGAACTCAAATCCTCTTTTGAGGAACAAGTTTTCTTGTCTATCGGCCT	
	TGCTGGTAATTCCTCTTTTGAGGAACAAGTTTTCTTGTATCCAGAACA	
	ATATTACCGCTCCTCTTTTGAGGAACAAGTTTTCTTGTCAGCCATTGC	
	AACAGGAAAATCCTCTTTTGAGGAACAAGTTTTCTTGTACGCTCATGG	
	AAATACCTACTCCTCTTTTGAGGAACAAGTTTTCTTGTATTTTGACGC	
	TCAATCGTCTTCCTCTTTTGAGGAACAAGTTTTCTTGTGAAATGGATT	
	ATTTACATTGTCCTCTTTTGAGGAACAAGTTTTCTTGTGCAGATTCAC	
	CAGTCACACGACCAGTAATAAAAGGGACAT	
B	TTACCTGAGCAAAAGAAGATGATGAAACAAACATCAAGAAAACA	52-57
	AAATTAATTATCCTCTTTTGAGGAACAAGTTTTCTTGTCATTTAACAA	
	TTTCATTTGATCCTCTTTTGAGGAACAAGTTTTCTTGTATTACCTTTT	
	TTAATGGAAATCCTCTTTTGAGGAACAAGTTTTCTTGTCAGTACATAA	
	ATCAATATATTCCTCTTTTGAGGAACAAGTTTTCTTGTGTGAGTGAAT	
	AACCTTGCTTTCCTCTTTTGAGGAACAAGTTTTCTTGTCTGTAAATCG	
	TCGCTATTAATCCTCTTTTGAGGAACAAGTTTTCTTGTTTAATTTTCC	
	CTTAGAATCCTCCTCTTTTGAGGAACAAGTTTTCTTGTTTGAAAACAT	
	AGCGATAGCTTCCTCTTTTGAGGAACAAGTTTTCTTGTTAGATTAAGA	
	CGCTGAGAAGAGTCAATAGTGAAT	
C	TGAATCTTACCAACGCTAACGAGCGTCTTTCCAGAGCCTAATTTGCCA GT	79-85
	TACAAAATAATCCTCTTTTGAGGAACAAGTTTTCTTGTACAGCCATAT	
	TATTTATCCCTCCTCTTTTGAGGAACAAGTTTTCTTGTAATCCAAATA	
	AGAAACGATTTCCTCTTTTGAGGAACAAGTTTTCTTGTTTTTGTTTAA	
	CGTCAAAAATTCCTCTTTTGAGGAACAAGTTTTCTTGTGAAAATAGCA	
	GCCTTTACAGTCCTCTTTTGAGGAACAAGTTTTCTTGTAGAGAATAAC	
	ATAAAAACAGTCCTCTTTTGAGGAACAAGTTTTCTTGTGGAAGCGCAT	
	TAGACGGGAGTCCTCTTTTGAGGAACAAGTTTTCTTGTAATTAACTGA	
	ACACCCTGAATCCTCTTTTGAGGAACAAGTTTTCTTGTCAAAGTCAGA	
	GGGTAATTGAGCGCTAATATCAGAGAGATAACCCACAAGAATTGAGTT AAGCCCAA	
D	TCACAAACAAATAAATCCTCATTAAAGCCAGAATGGAAAGCGCAGTCT CTGAATTT	106-112
	ACCGTTCCAGTCCTCTTTTGAGGAACAAGTTTTCTTGTTAAGCGTCAT	
	ACATGGCTTTTCCTCTTTTGAGGAACAAGTTTTCTTGTTGATGATACA	
	GGAGTGTACTTCCTCTTTTGAGGAACAAGTTTTCTTGTGGTAATAAGT	
	TTTAACGGGGTCCTCTTTTGAGGAACAAGTTTTCTTGTTCAGTGCCTT	
	GAGTAACAGTTCCTCTTTTGAGGAACAAGTTTTCTTGTGCCCGTATAA	
	ACAGTTAATGTCCTCTTTTGAGGAACAAGTTTTCTTGTCCCCCTGCCT	
	ATTTCGGAACTCCTCTTTTGAGGAACAAGTTTTCTTGTCTATTATTCT	
	GAAACATGAATCCTCTTTTGAGGAACAAGTTTTCTTGTAGTATTAAGA	
	GGCTGAGACTCCTCAAGAGAAGGATTAGGATTAGCGGGGTTTTGCTCA GT	
E	CAAAGTACAACGGAGATTTGTATC	134-139
	ATCGCCTGATTCCTCTTTTGAGGAACAAGTTTTCTTGTAAATTGTGTC	
	GAAATCCGCGTCCTCTTTTGAGGAACAAGTTTTCTTGTACCTGCTCCA	
	TGTTACTTAGTCCTCTTTTGAGGAACAAGTTTTCTTGTCCGGAACGAG	
	GCGCAGACGGTCCTCTTTTGAGGAACAAGTTTTCTTGTTCAATCATAA	
	GGGAACCGAATCCTCTTTTGAGGAACAAGTTTTCTTGTCTGACCAACT	
	TTGAAAGAGGTCCTCTTTTGAGGAACAAGTTTTCTTGTACAGATGAAC	
	GGTGTACAGATCCTCTTTTGAGGAACAAGTTTTCTTGTCCAGGCGCAT	
	AGGCTGGCTGTCCTCTTTTGAGGAACAAGTTTTCTTGTACCTTCATCA	
	AGAGTAATCTTGACAAGAACCGGATATTCATTACCCAAATCAAC	
F	ACAGTTGATTCCCAATTCTGCGAACGAGTA	161-165
	GATTTAGTTTTCCTCTTTTGAGGAACAAGTTTTCTTGTGACCATTAGA	
	TACATTTCGCTCCTCTTTTGAGGAACAAGTTTTCTTGTAAATGGTCAA	
	TAACCTGTTTTCCTCTTTTGAGGAACAAGTTTTCTTGTAGCTATATTT	
	TCATTTGGGGTCCTCTTTTGAGGAACAAGTTTTCTTGTCGCGAGCTGA	
	AAAGGTGGCATCCTCTTTTGAGGAACAAGTTTTCTTGTTCAATTCTAC	
	TAATAGTAGTTCCTCTTTTGAGGAACAAGTTTTCTTGTAGCATTAACA	

	TCCAATAAATTCCTCTTTTGAGGAACAAGTTTTCTTGTCATACAGGCA	
	AGGCAAAGAATCCTCTTTTGAGGAACAAGTTTTCTTGTTTAGCAAAAT	

Table S4. Reported SNR values of glass and SiN_x nanopores in the literature.

Pore Type	Targets	Pore Diameter	Pore Length	Cut-off Frequency	Voltage	SNR
SiN_x[3]	30 nt ssDNA	1.4 nm	5 nm	1 MHz	1000 mV	37
Glass[4]	proteins	8 nm		100 kHz	1000 mV	9
SiN_x[5]	40 nt ssDNA	2.5 nm	5 nm	10 kHz	200 mV	47.5
SiN_x[6]	100 nt ssDNA	2 nm	3 nm	200 kHz	900 mV	10
SiN_x[7]	50 bp DNA	5 nm	15 nm	1 MHz	500 mV	10
Glass[8]	λDNA	14 nm		10 kHz	500 mV	25
SiN_x[1]	λDNA	30 nm	20 nm	10 kHz	300 mV	22
Glass[9]	10 kbp DNA	40 nm		10 kHz	600 mV	7
SiN_x This work	proteins	5 nm	15 nm	50 kHz	100 mV	121
Glass This work	7.2 kbp dsDNA	5 nm		50 kHz	500 mV	81

Table S5. Boundary conditions used in the FEM simulations for the two nanopore platforms.

Scheme	Surface	Poisson	Nernst-Planck	Navier-Stokes
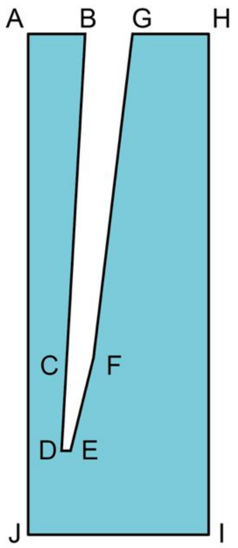	**AB**	Constant potential $\phi = V_{app}$	Constant concentration $c_i = C_{bulk}$	Constant pressure $p = 0$ no viscous stress $\mathbf{n} \cdot [\mu(\nabla \mathbf{u} + (\nabla \mathbf{u})^\mathsf{T})] = 0$
	**DE, EF, FG, GH, HI**	no charge $-\mathbf{n} \cdot (\varepsilon \nabla \phi) = 0$	no flux $n \cdot N_i = 0$	no slip
	**IJ**	Constant potential $\phi = 0$	Constant concentration $c_i = C_{bulk}$	Constant pressure $p = 0$ no viscous stress $\mathbf{n} \cdot [\mu(\nabla \mathbf{u} + (\nabla \mathbf{u})^\mathsf{T})] = 0$
	**AJ**	axial symmetry	axial symmetry	axial symmetry
	**BC, CD**	$-\mathbf{n} \cdot (\varepsilon \nabla \phi) = \sigma_w$	no flux $n \cdot N_i = 0$	no slip
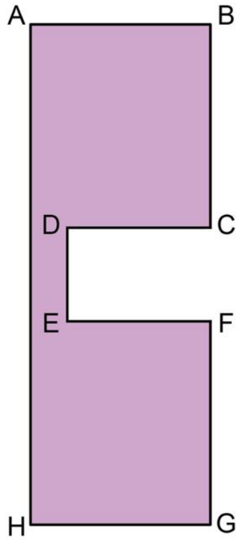	**AB**	Constant potential $\phi = V_{app}$	Constant concentration $c_i = C_{bulk}$	Constant pressure $p = 0$ no viscous stress $\mathbf{n} \cdot [\mu(\nabla \mathbf{u} + (\nabla \mathbf{u})^\mathsf{T})] = 0$
	**BC, CD, EF, FG**	no charge $-\mathbf{n} \cdot (\varepsilon \nabla \phi) = 0$	no flux $n \cdot N_i = 0$	no slip
	**GH**	Constant potential $\phi = 0$	Constant concentration $c_i = C_{bulk}$	Constant pressure $p = 0$ no viscous stress $\mathbf{n} \cdot [\mu(\nabla \mathbf{u} + (\nabla \mathbf{u})^\mathsf{T})] = 0$
	**AH**	axial symmetry	axial symmetry	axial symmetry
	**DE**	$-\mathbf{n} \cdot (\varepsilon \nabla \phi) = \sigma_w$	no flux $n \cdot N_i = 0$	no slip

Table S6. Fabrication and usability features comparison (Glass vs. SiN_x nanopores). This comparison is based on glass nanopores fabricated by the laser-pulling method and SiN_x nanopores fabricated by TEM drilling.

Feature	Glass Nanopores	SiN_x Nanopores
Fabrication Complexity	Straightforward production by laser-assisted pulling of glass capillaries	Requires instrumentation and expertise for lithographic patterning and beam-based drilling
Fabrication Repeatability	Highly repeatable for > 5 nm nanopores	Highly repeatable for > 5 nm nanopores
Yield	~1 nanopore/min	~1 nanopore/20 min
Wetting/Filling Reliability	Challenging for pores smaller than 5 nm	Good hydrophilicity after piranha solution treatment
Usable Lifetime	~ 19 weeks if properly stored[10]	Reusable but pore expands at ~ 0.2 to 3 nm/day[11]
Device-Handling Constraints	restricted access to the trans reservoir, complicating buffer exchange and sample handling	Straightforward access to both cis and trans reservoirs
Stability	Generally good but capillary tips may break during handling	Generally good but SiN_x membrane may break during filling and cleaning

References:

1. Liu, W.; Zhang, Y.; Gu, Z.; et al. *IEEE Sens. J.* **2024**, *24* (3), 2405–2412.
2. van den Hout, M.; Hall, A. R.; Wu, M. Y.; et al. *Nanotechnology* **2010**, *21* (11), 115304.
3. Venta, K.; Shemer, G.; Puster, M.; et al. *ACS Nano* **2013**, *7* (5), 4629–4636.
4. Bandara, Y. M. N. D. Y.; Freedman, K. J. *ACS Nano* **2022**, *16* (9), 14111–14120.
5. Lee, M.-H.; Kumar, A.; Park, K.-B.; et al. *Sci Rep* **2014**, *4* (1), 7448.
6. Shekar, S.; Niedzwiecki, D. J.; Chien, C.-C.; et al. *Nano Lett.* **2016**, *16* (7), 4483–4489.
7. Rosenstein, J. K.; Wanunu, M.; Merchant, C. A.; et al. *Nat. Methods* **2012**, *9* (5), 487–492.
8. Steinbock, L. J.; Bulushev, R. D.; Krishnan, S.; et al. *ACS Nano* **2013**, *7* (12), 11255–11262.
9. Cadinu, P.; Kang, M.; Nadappuram, B. P.; et al. *Nano Lett.* **2020**, *20* (3), 2012–2019.
10. Alawami, M. F.; Bošković, F.; Zhu, J.; et al. *iScience* **2022**, *25* (5), 104191.
11. Chou, Y.-C.; Masih Das, P.; Monos, D. S.; et al. *ACS Nano* **2020**, *14* (6), 6715–6728.
